# Prevalence of permanent childhood hearing loss detected at the universal newborn hearing screen: Systematic review and meta-analysis

**DOI:** 10.1371/journal.pone.0219600

**Published:** 2019-07-11

**Authors:** Emma Butcher, Carol Dezateux, Mario Cortina-Borja, Rachel L. Knowles

**Affiliations:** 1 Life Course Epidemiology and Biostatistics, UCL Great Ormond Street Institute of Child Health, London, United Kingdom; 2 Centre for Primary Care and Public Health, Barts and the London School of Medicine and Dentistry, Queen Mary University of London, London, United Kingdom; 3 Clinical Epidemiology, Nutrition and Biostatistics, UCL Great Ormond Street Institute of Child Health, London, United Kingdom; 4 Public Health England, London, United Kingdom; Universitat Witten/Herdecke, GERMANY

## Abstract

**Context:**

Permanent childhood hearing loss (PCHL) can affect speech, language, and wider outcomes. Adverse effects are mitigated through universal newborn hearing screening (UNHS) and early intervention.

**Objective:**

We undertook a systematic review and meta-analysis to estimate prevalence of UNHS-detected PCHL (bilateral loss ≥26 dB HL) and its variation by admission to neonatal intensive care unit (NICU). A secondary objective was to report UNHS programme performance (PROSPERO: CRD42016051267).

**Data sources:**

Multiple electronic databases were interrogated in January 2017, with further reports identified from article citations and unpublished literature (November 2017).

**Study selection:**

UNHS reports from very highly-developed (VHD) countries with relevant prevalence and performance data; no language or date restrictions.

**Data extraction:**

Three reviewers independently extracted data and assessed quality.

**Results:**

We identified 41 eligible reports from 32 study populations (1799863 screened infants) in 6195 non-duplicate references. Pooled UNHS-detected PCHL prevalence was 1.1 per 1000 screened children (95% confidence interval [CI]: 0.9, 1.3; *I*^*2*^ = 89.2%). This was 6.9 times (95% CI: 3.8, 12.5) higher among those admitted to NICU. Smaller studies were significantly associated with higher prevalences (Egger’s test: *p* = 0.02). Sensitivity and specificity ranged from 89–100% and 92–100% respectively, positive predictive values from 2–84%, with all negative predictive values 100%.

**Limitations:**

Results are generalisable to VHD countries only. Estimates and inferences were limited by available data.

**Conclusions:**

In VHD countries, 1 per 1000 screened newborns require referral to clinical services for PCHL. Prevalence is higher in those admitted to NICU. Improved reporting would support further examination of screen performance and child demographics.

## Introduction

Universal newborn hearing screening (UNHS) programmes enable prompt detection and intervention for early-onset permanent childhood hearing loss (PCHL). This facilitates improved speech and language development, as well as better health, educational and social outcomes [1, 2]. Most very highly-developed (VHD) countries [3] have now implemented UNHS programmes, defined as universal screening by age 6 months with otoacoustic emissions (OAE) tests, auditory brainstem response (ABR) tests, or both, followed by diagnostic referral where indicated [4].

Implemented programmes vary in size, programme quality, performance, and reported prevalence of PCHL. The latter may be attributable to differences in type, timing, and frequency of test procedures, referral criteria, diagnostic case definition, and management of at-risk children (usually defined according to Joint Committee on Infant Hearing [JCIH] criteria [1] or by neonatal intensive care unit [NICU] admission) [4–8]. Methods of follow-up to ascertain PCHL among those with negative screening results (i.e. children that are not referred to diagnostic testing) can also play an important role (for example, targeted surveillance or subsequent screening).

Previous reviews of UNHS programmes have evaluated screening test methods or quality, and timing of intervention after PCHL diagnosis [9–11]. There has been little quantitative synthesis of PCHL prevalence or analysis of heterogeneity, particularly relating to demographic and individual differences in the populations screened. Additionally, performance of entire UNHS programme pathways as oppose to single tests (relating to measures of accuracy including sensitivity, specificity, negative predictive value, and positive predictive value) have not been systematically examined. Addressing these research gaps will inform screening policy and improve service planning for children with PCHL, by enabling evaluation of UNHS programmes in respect to key indicators and benchmarks. Estimating the prevalence of PCHL detected through UNHS also permits examination of secular trends and heterogeneity in terms of population and UNHS programme characteristics. This provides vital information on UNHS programme performance and the effectiveness of PCHL prevention efforts.

We carried out a systematic review and meta-analysis to estimate the prevalence of PCHL (defined as bilateral PCHL ≥26 dB HL confirmed by diagnostic tests) detected through UNHS in VHD countries. Our secondary objectives were to examine how detected PCHL prevalence varies between studies and by demographic characteristics, as well as to estimate UNHS programme performance.

## Methods

The review was carried out in accordance with the registered PROSPERO protocol (CRD42016051267) and reported following MOOSE (S1 Table) and PRISMA (S2 Table) guidelines.

### Search strategy

To identify eligible studies, in January 2017 one reviewer (EB) interrogated electronic databases (PubMed, Medline(OvidSP), EMBASE, CINAHL, and the Cochrane Library) and reviewed the first 100 Google Scholar search results. Further reports were identified from citations of included papers and unpublished literature (November 2017). Text-word searches, along with MeSH terms or Subject Headings, were used to construct database searches. Key text-words related to: hearing loss, hearing impairment, deafness, epidemiology, incidence, prevalence, and newborn, neonatal, child, infant, etc. (S1 File). There were no date or language restrictions; all published reports were considered for inclusion if there was an English abstract. Searches of unpublished literature included relevant screening programme reports in any language, whether or not they had English abstracts.

### PCHL definition

The review case definition for PCHL was bilateral PCHL ≥26 dB HL. This reflects the minimum severity of PCHL defined by WHO that is expected to require long-term active management [12] and excludes temporary conditions. Although milder (15–26 dB HL) and unilateral PCHL can also impact on outcomes [13], these were not included as they are not always detected, or systematically reported, by UNHS programmes. Acquired, progressive or late-onset conditions were not included as UNHS does not capture these.

### Inclusion and exclusion criteria

We included reports of programmes from VHD countries as defined by the United Nations Development Programme (UNDP), such as the United Kingdom, United States, and Germany, [3], as these are similar to each other in terms of UNHS provision, access to health care, and child health and socioeconomic conditions. Studies were included if an English abstract was available (not applicable to unpublished reports), UNHS was in place during the study, and the total number of children with UNHS-detected PCHL fitting the review case definition was reported, as well as the total number considered for, or undergoing, UNHS.

Studies were excluded if any inclusion criteria were not met, the minimum threshold for PCHL exceeded 61 dB HL, they were an ineligible study or article type (review without a systematic search strategy, comment piece, letter, or editorial), there was evidence of ascertainment bias, or all participants were aged over 1 year by study start (as UNHS should occur before age 6 months). As the aim was to estimate population-based prevalence, studies involving a selective sample of children considered to be at high-risk of PCHL were excluded, whilst those including only children at low-risk of PCHL were included.

### Article selection, data extraction, and quality evaluation

One reviewer (EB) screened titles and abstracts of all identified reports against the inclusion and exclusion criteria. A second reviewer (RK or CD) each reviewed a random 10% sample, with inter-rater concordance assessed by calculation of the unweighted kappa statistic; discrepancies were resolved by discussion. EB screened full reports against inclusion and exclusion criteria; uncertainties were discussed with RK and CD.

Two reviewers (EB with either RK or CD) independently extracted data for each included study using a form piloted on several studies before use (S2 File). Each study was classified into one of four geographical regions based on included countries: Asia, Europe, North America, and Australia. This form included questions regarding quality based on the JCIH guidelines [1], Newcastle-Ottawa scale [14], STARD [15], and QUADAS-2 [16] criteria. Studies were scored against eight quality criteria, with a maximum of nine points available (Table 1), as well as by the original QUADAS-2 criteria. We resolved discrepancies by discussion. Study data were collected and managed using REDCap (Research Electronic Data Capture), a web-based application for data collection, hosted at University College London [17].

**Table 1:** Quality scoring criteria.

Factor	Score	Scoring
**PCHL definition**	0	Not clearly defined—missing information on >1 feature (laterality, anatomy and severity of study target condition)
	1	Mostly defined—missing information on 1 feature
	2	Clearly defined—specified all features
**Other concerns**	0	Further concern identified
	1	No other concerns identified
**Clear protocol**	0	Not clearly defined–missing information on one or more features (which tests and number of stages)
	1	Clearly defined
**Clear at-risk protocol**	0	Not clearly defined (how at-risk infants, as defined by the study, were managed)
	1	Clearly defined
**Sample bias**	0	Any identified concerns
	1	No identified concerns
**UNHS coverage**	0	<95% or unclear (# receiving ≥1 UNHS tests) / (target population)
	1	≥95%
**UNHS follow-up**	0	<70% or unclear (# not lost to follow-up by end of diagnostic tests) / (# failing first stage of UNHS)
	1	≥70%
**Overall follow-up**	0	No follow-up after completion of relevant screening tests (screen negatives) or diagnostic testing (screen positives)
	1	Yes, some form of follow-up reported

PCHL: permanent childhood hearing loss; UNHS: universal newborn hearing screening.

### Statistical analysis plan

We included each study sample in analyses only once, thus multiple reports from single or overlapping study populations were combined where possible; when indicated, the report providing most detail was used with related papers included as additional references.

We evaluated the characteristics of included studies. We calculated, for each study, the prevalence and 95% confidence intervals (CI) of children with PCHL detected via UNHS and fitting the review case definition in the screened population (defined as the number of children receiving one or more screening tests). Pooled PCHL prevalence and 95% CIs were calculated using the Freeman-Tukey double arc-sine transformation of proportions and Wilson (Score) method [18]. Random-effects models using the Der Simonian and Laird method [19] were fitted to account for the expected heterogeneity in the screened populations and screening methods used. Heterogeneity was evaluated with the *I*^*2*^ statistic (proportion of observed variance not explained by chance) [20] and explored by stratifying prevalence calculations by study characteristics. Sensitivity analyses were carried out excluding outliers and studies of low quality. Funnel plots and Egger’s test were used to assess small-sample bias, along with a sensitivity analysis excluding studies with fewer than 7832 children (based on minimum sample size required to accurately detect a prevalence of 1 per 1000 children within 95% CIs and a precision of 0.0005) [21]. Significance tests were conducted at 5% level.

Screening programme performance was assessed using available data on programme outcomes, namely number of children with screen positive and negative results, and with (true positives, false negatives) or without (false positives, true negatives) confirmed diagnoses. Screen positives comprised all children referred to diagnostic testing, and screen negatives all those not referred, regardless of attendance at diagnostic testing and attrition before the point of diagnostic referral. True positives were defined as all screen-positive children diagnosed with PCHL fitting the review case definition, whilst false positives were all screen-positive children that did not have PCHL fitting the review case definition. We only calculated negative predictive value (NPV), sensitivity and specificity for studies with follow-up to ascertain false negatives (excluding unscreened children with PCHL, plus late-onset, acquired and progressive PCHL from the false negative number where possible). Positive predictive value (PPV) calculation was not limited by follow-up. Quantitative pooling of performance estimates was not undertaken due to methodological differences between studies, such as tests used or diagnostic referral criteria for testing.

PCHL prevalence by reported demographic and individual characteristics and in those with or without NICU admission were explored and calculated where data were available. Analyses were performed using STATA 15 (Stata Corp, College Station, TX).

## Results

### Selection of eligible studies

**Fig 1:**
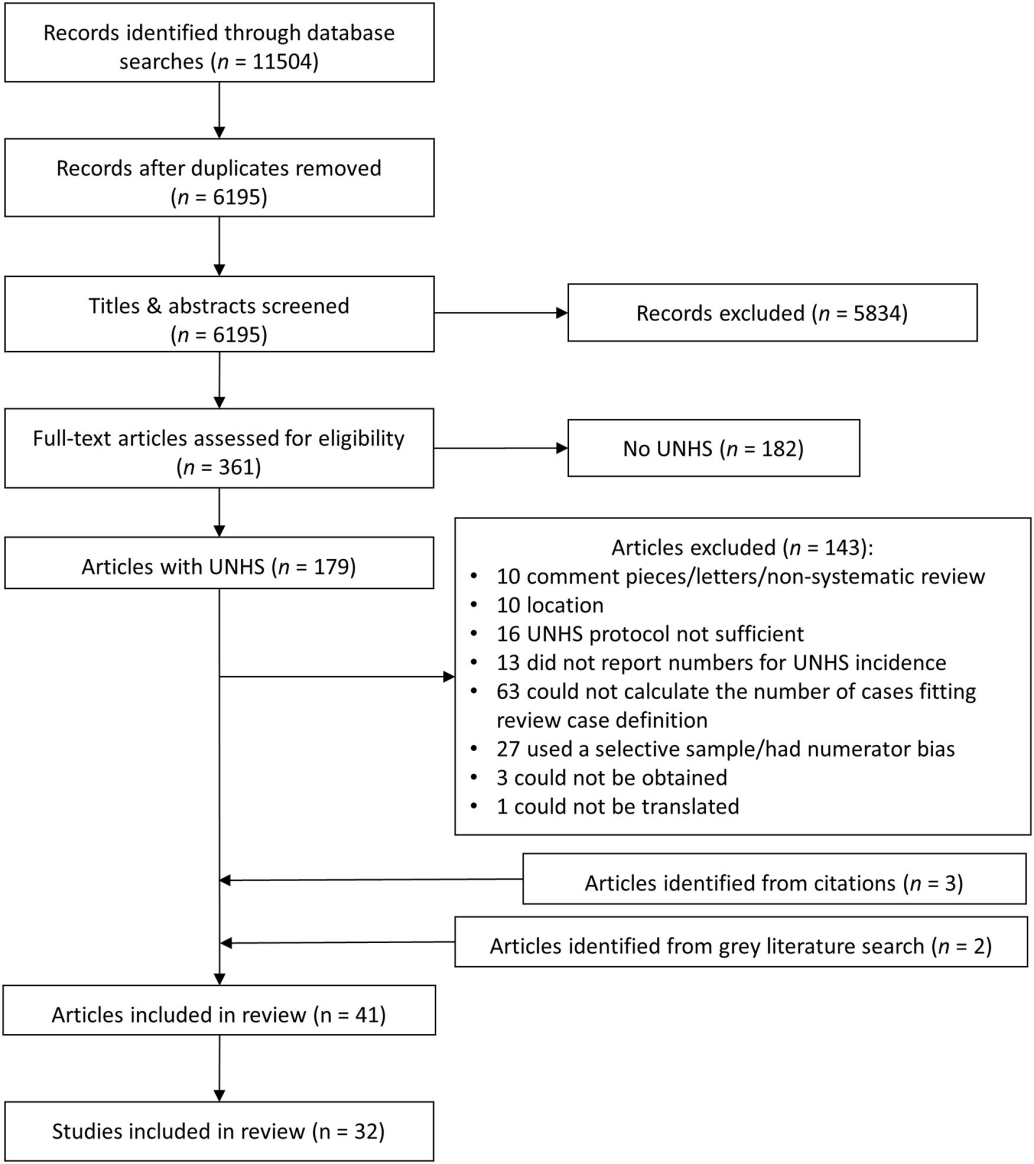
PRISMA flow diagram.

The literature search identified 6195 non-duplicate records, of which 5834 were excluded at the title and abstract screen. A further 325 records were excluded after screening full texts. Five relevant articles were identified from searches of the grey literature and references of included papers. This resulted in 41 articles for inclusion in the meta-analysis, reporting on 32 separate study populations (Fig 1). Two systematic reviews [9, 10] were included in the total number of articles, however these were not included in data extraction as they contained no additional data (all reviewed studies were already included as individual studies).

The summary unweighted inter-rater kappa statistic for abstract inclusion were 0.8 for EB with both RK and CD, implying good agreement. Reviewers agreed the final list of eligible studies by consensus, therefore inter-rater differences were not assessed.

### Study characteristics

The characteristics of the included studies are described in Table 2, separately for those with and without follow-up after screening and diagnostic testing was completed. Over 70% of included studies were from Europe (*n* = 23, 72%), with the remainder from North America (*n* = 4, 13%), Australia (*n* = 3, 9%), or Asia (*n* = 2, 6%). Seven studies aimed to include out-of- hospital births (i.e. were ‘population-based’), whilst 12 involved a single hospital, and 13 multiple hospitals or birth centres. The Wessex study involved four hospitals that alternated UNHS and non-UNHS screening periods; only data reported from UNHS screening periods are considered here.

**Table 2:** Included study characteristics.

Study authors	Location	Setting	Period of UNHS	UNHS protocol	Reported PCHL meeting review case definition	Study population, *n* (% screened)	PCHL diagnoses via UNHS, *n*	Follow-up after UNHS and diagnostic testing
**Studies with follow-up**								
**Almenar Latorre** [22]	Spain	1 H	Unclear	OAE & ABR	Bilateral >40 dB HL^a^	1532 (n/a)^e^	4	Parent questionnaire at age 1 year (n = 825, 53.8%, followed up)
**Antoni** [23]	France	4 H	2005–2010	ABR	Bilateral >35 dB HL^a^	27885 (96.0)	30	Children with PCHL diagnoses via UNHS had audiology records checked (mean follow-up length = 34 months, range 0–75 months)
**Berninger** [24]	Sweden	2 H	1999–2004	OAE & ABR	Bilateral SNHL, CHL & mixed >30 dB HL^b^	31092 (n/a)^e^	57	Clinician report of further cases and acoustic amplification referral database checked up to age 10 years
**Calcutt** [25]	Australia	P—1 region	2009–2011	ABR	Bilateral SNHL >40 dB HL^b^	185205 (94.9)	121	Targeted surveillance screen at 9–12 months (2606 attended of 4361 referred) and audiology records checked up to age 4 years
**Calevo** [26]	Italy	13 H/BC	2002–2004	OAE & ABR	Bilateral >40 dB HL^a^^,^^b^^,^^c^	32502 (99.2)	20	PCHL registry and audiology records checked up to age 2 years
**Cao-Nguyen** [27]	Switzerland	1 H	2000–2004	OAE & ABR	Bilateral >40 dB HL^a^^,^^d^	17535 (n/a)^e^	24	Audiology records checked to unclear age (excluding late-onset PCHL)
**De Capua** [28]	Italy	3 H	1998–2007	OAE	Bilateral SNHL, CHL & mixed >30 dB HL^b^	21125 (93.3)	24	Audiology records checked up to age 1 year
**Ng** [29]	Hong Kong	1 H	1999–1999	OAE	Bilateral ≥40 dB HL^a^^,^^b^	1076 (98.9)	3	Parent interviews at 18 and 36 months (n = 1020; 95.9% followed up)
**O'Connor** [30]	Ireland	6 H	2011–2012	OAE & ABR	Bilateral SNHL, CHL & mixed >40 dB HL^b^^,^^c^	11763 (99.8)	12	Targeted surveillance screen up to age 1 year
**Watkin** [31–33]	UK	P—1 region	1992–2002	OAE	Bilateral ≥40 dB HL^a^^,^^b^^,^^c^	35668 (94.9)	32	Audiology records checked up to age 12 years (excluding late-onset PCHL)
**Wessex** [34–36]	UK	4 H—only periods with UNHS included	1993–1996	OAE & ABR	Bilateral ≥40 dB HL^a^	25609 (83.1)	22	Active follow-up and audiology, healthcare and educational records checked up to age 9 years (excluding late-onset PCHL)
**Studies without follow-up**								
**Adelola** [37]	Ireland	2 H	2000–2007	OAE & ABR	Bilateral >40 dB HL^a^^,^^b^	26281 (97.9)	19	
**Aidan** [38]	France	1 H	1995–1997	OAE	Bilateral SNHL >40 dB HL^b^	1727 (82.3)	2	
**Bailey** [39]	Australia	5 H–excluding some NICU infants	2000–2001	OAE & ABR	Bilateral >35 dB HL^a^^,^^b^	13214 (96.2)	5	
**Caluraud** [40]	France	14 H/BC	1999–2011	OAE & ABR	Bilateral >35 dB HL^a^^,^^d^	101916 (99.8)	142	
**Fornoff** [41]	USA	P—Statewide	2003–2004	OAE & ABR	Bilateral SNHL, CHL & mixed ≥30 dB HL^b^	335412 (98.0)	160	
**Ghirri** [42]	Italy	1 H	2005–2009	OAE & ABR	Bilateral >40 dB HL^a^^,^^b^^,^^c^	8113 (n/a)^e^	21	
**Gonzalez de Aledo Linos** [43, 44]	Spain	2 H	2001–2003	OAE	Bilateral SNHL >40 dB HL^b^	8836 (98.4)	11	
**Guastini** [45]	Italy	1 H	2006–2009	OAE & ABR	Bilateral >40 dB HL^a^^,^^b^^,^^d^	8671 (n/a)^e^	2	
**Habib** [46]	Saudi Arabia	1 H—excluding children with JCIH 1994 risk factors	1996–2004	OAE	Bilateral SNHL ≥26 dB HL^b^	11986 (n/a)^e^	20	
**Magnani** [47]	Italy	1 H	2010–2013	OAE & ABR	Bilateral SNHL >40 dB HL^b^^,^^c^	11624 (99.7)	26	
**Martinez** [48]	Spain	1 H	2001–2002	OAE & ABR	Bilateral >35 dB HL^a^^,^^b^	1277 (94.2)	9	
**Mason** [49]	USA	1 H	1992–1997	ABR	Bilateral SNHL, CHL & mixed >35 dB HL^b^	10773 (98.2)	16	
**Mehl** [50]	USA	57 H	1992–1999; only 1999 included	ABR	Bilateral SNHL, CHL & mixed ≥35 dB HL^b^	63590 (87.0)	63	
**Metzger** [51]	Switzerland	1 H—excluding preterm births	2005–2010	OAE	Bilateral ≥40 dB HL^a^	12080 (n/a)	15	
**NSW** [52]	Australia	P– 1 region	2003–2009	ABR	Bilateral >40 dB HL^a^^,^^b^	284694 (99.0)	283	
**Rohlfs** [53]	Germany	14 H/BC	2002–2006	OAE & ABR	Bilateral ≥41 dB HL^a^^,^^b^^,^^c^	65466 (92.8)	73	
**Uilenburg** [54]	Netherlands	P—3 regions, excluding NICU infants	1999–2000	OAE	Bilateral SNHL ≥40 dB HL^b^	3336 (94.0)	1	
**Uus** [55]	UK	23 H	2001–2004	OAE & ABR	Bilateral SNHL, CHL & mixed ≥40 dB HL	169487 (n/a)^e^	169	
**Van der Ploeg** [56]	Netherlands	P—entire country, excluding NICU infants	2002–2009 (excluding 2007)	OAE & ABR	Bilateral ≥40 dB HL^a^^,^^b^	552820 (n/a)	427	
**Van Kerschaver** [6, 57, 58]	Belgium	P—1 region	1999–2008	ABR	Bilateral SNHL, CHL & mixed >40 dB HL^b^	628337 (95.9)	646	
**White** [59]	USA	1 H—random sample	1990–1991	OAE & ABR	Bilateral SNHL, CHL & mixed >25 dB HL^b^	1850 (n/a)^e^	6	

^a^Anatomy of HL (CHL, SNHL, mixed) not specified.

^b^Unilateral loss also reported in study, but excluded in meta-analysis PCHL count.

^c^PCHL <40 dB HL also reported but <26 dB HL and 26–40 dB HL PCHL could not be separated.

^d^Diagnostic threshold unclear; assumed to be the same as the screening test threshold.

^e^Unclear study population considered; number given is those receiving ≥1 UNHS test.

ABR: auditory brainstem response; BC: birth clinic; CHL: conductive HL; dB HL: decibels hearing level; H: hospital; n/a: not applicable: NICU: neonatal intensive care unit; OAE: otoacoustic emissions; P: population-based (including out of hospital births); PCHL: permanent childhood hearing loss; SNHL: sensorineural HL; UNHS: universal newborn hearing screening.

Median study duration was 3 years (25^th^ percentile [Q1]: 2, 75^th^ percentile [Q3]: 6), with included studies covering births between 1990 and 2014. Results from 1999 for the Mehl study [50] are included as the numbers screened and diagnosed were unclear for other years.

Study population ranged between 1076 and 628337 (median 25945, Q1: 11198, Q3: 83691) children for the 24 studies with this information available. Median study population recruitment or screening coverage was 96.2% (Q1: 94.2, Q3: 98.9%) for 21 studies specifying both the total study population and the number screened, with a total of 1799863 screened children in the included studies.

UNHS screen protocol types were broadly grouped to: OAE-only (8 studies), ABR-only (6 studies), or OAE and ABR (18 studies), based on the screening tests used in the main study protocol (S3 Table).

### PCHL diagnoses

Between 1 and 646 children were diagnosed with PCHL fitting the review case definition via UNHS in the individual studies. Most studies did not report follow-up after completion of UNHS and diagnostic testing (*n* = 21). Of the 11 studies with any follow-up, nine attempted to follow-up the entire study population, one only reported targeted surveillance results for screen negative children with risk factors (O’Connor) [30] and one study only followed up true positive children (Antoni) [23]. Thus there were 10 studies with follow-up of screen negative children. Methods of follow-up for screen negatives involved checking for PCHL diagnoses via local clinicians, audiology databases, registers or other records (*n* = 5), interviewing parents (*n* = 2), targeted surveillance (*n* = 2; with one study also assessing audiology records), and active testing along with checking for further diagnoses from multiple health, education and audiology databases and staff involved in management of children with PCHL (*n* = 1) (Table 2).

### Study quality

**Fig 2:**
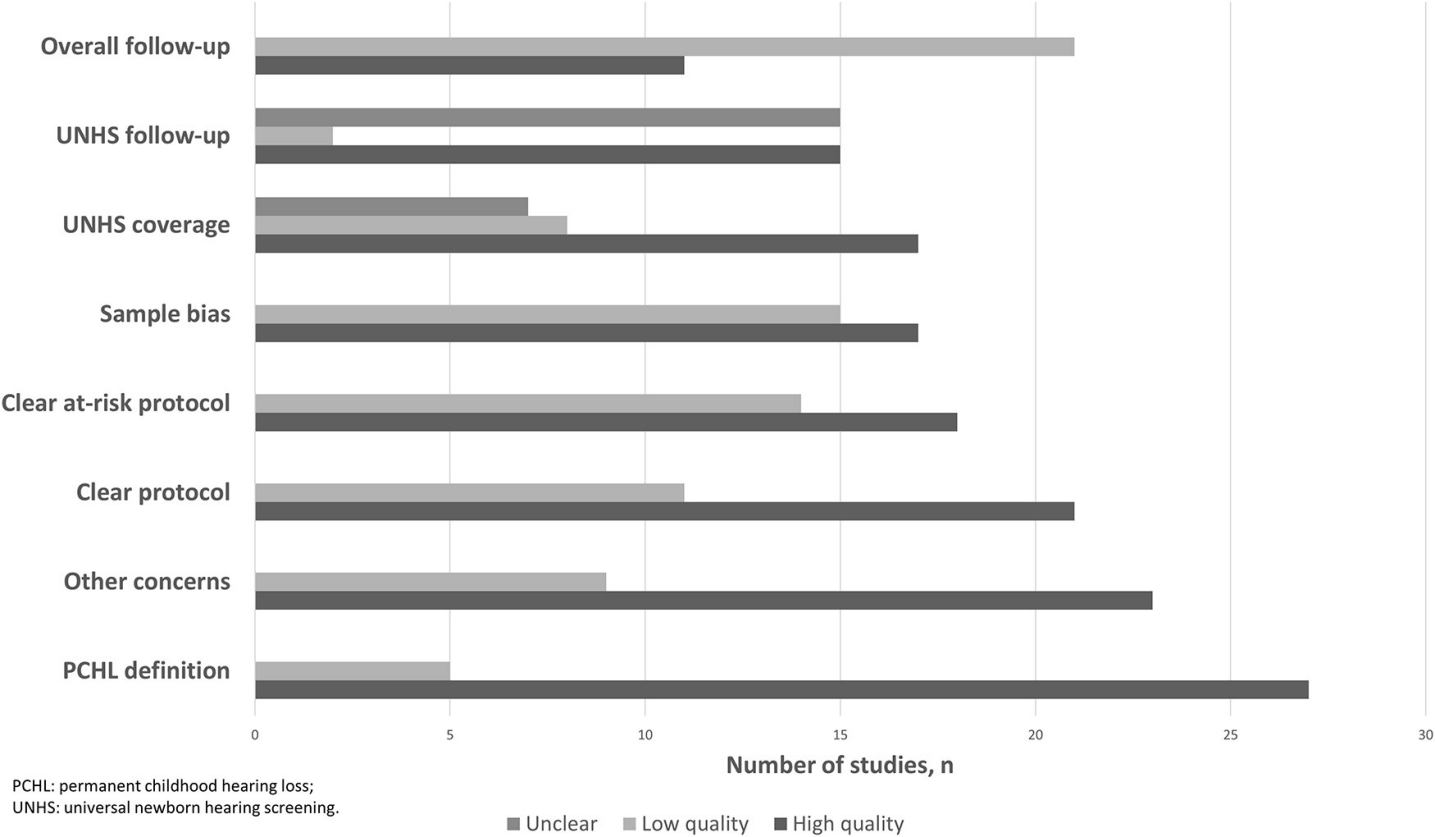
Study quality indicators.

The majority of studies were of high quality for the following criteria: clarity of PCHL case definition, protocol details, clear description for management of at-risk children, lack of sample bias, high UNHS coverage (≥95%), and absence of other identified concerns. Lower or more variable quality was seen for UNHS follow-up, as 15 studies did not clearly state the number of children lost to follow-up during the UNHS and diagnostic testing stages. Additionally, few studies reported any follow-up after screening and diagnostic testing was completed (Table 3; Fig 2). Those with follow-up usually relied on passive methods, as described above, and rarely reported whether screen-negative children with later diagnoses were failures of detection (false negatives) or had late-onset, acquired or progressive PCHL. Results of QUADAS-2 scoring (relating specifically to risk of bias) are presented in S3 File.

**Table 3:** UNHS meta-analysis study quality scoring results.

Study	PCHL definition	Other concerns	Clear protocol	Clear at-risk protocol	Sample bias	UNHS coverage	UNHS follow-up	Overall follow-up
De Capua	2	1	1	1	1	0	1	1
O'Connor	2	1	1	1	1	1	n/a	1
Calcutt	2	1	1	1	0	1	n/a	1
Calevo	0	1	1	1	1	1	1	1
Magnani	2	1	0	1	1	1	1	0
Mason	2	1	1	1	0	1	1	0
Ng	1	0	1	1	1	1	1	1
Caluraud	0	1	1	1	1	1	1	0
Guastini	1	1	1	1	1	n/a	1	0
Uilenburg	2	1	1	1	0	0	1	0
Uus	2	1	1	1	1	n/a	n/a	0
White	2	1	1	1	0	n/a	1	0
Almenar Latorre	1	0	1	1	0	n/a	1	1
Gonzalez de Aledo Linos	1	1	1	0	1	1	n/a	0
Habib	2	1	1	0	0	0	1	0
NSW	1	1	0	0	1	1	1	0
Van der Ploeg	1	1	1	1	0	1	n/a	0
Watkin	1	1	0	0	1	1	n/a	1
Wessex	1	1	1	1	0	0	n/a	1
Adelola	1	0	0	1	1	1	n/a	0
Antoni	1	0	0	0	0	1	1	1
Bailey	1	0	1	0	0	1	0	1
Cao-Nguyen	0	1	0	0	1	1	n/a	1
Ghirri	1	1	1	1	0	n/a	n/a	0
Metzger	1	1	1	0	0	n/a	1	0
Berninger	2	0	0	0	0	n/a	n/a	1
Fornoff	1	0	0	0	1	1	n/a	0
Martinez	1	1	0	0	1	0	n/a	0
Mehl	2	0	0	0	1	0	n/a	0
Rohlfs	0	1	1	1	0	0	0	0
Van Kerschaver	1	0	0	0	1	1	n/a	0
Aidan	0	1	1	0	0	0	0	0
**Total high quality**	**11 high,** **16 medium**	**23**	**21**	**18**	**17**	**17**	**15**	**11**
**Total low quality**	**5**	**9**	**11**	**14**	**15**	**8**	**2**	**21**
**Unclear**	**n/a**	**n/a**	**n/a**	**n/a**	**n/a**	**7**	**15**	**n/a**

n/a indicates unclear quality. PCHL: permanent childhood hearing loss; UNHS: universal newborn hearing screening.

### Prevalence

**Fig 3:**
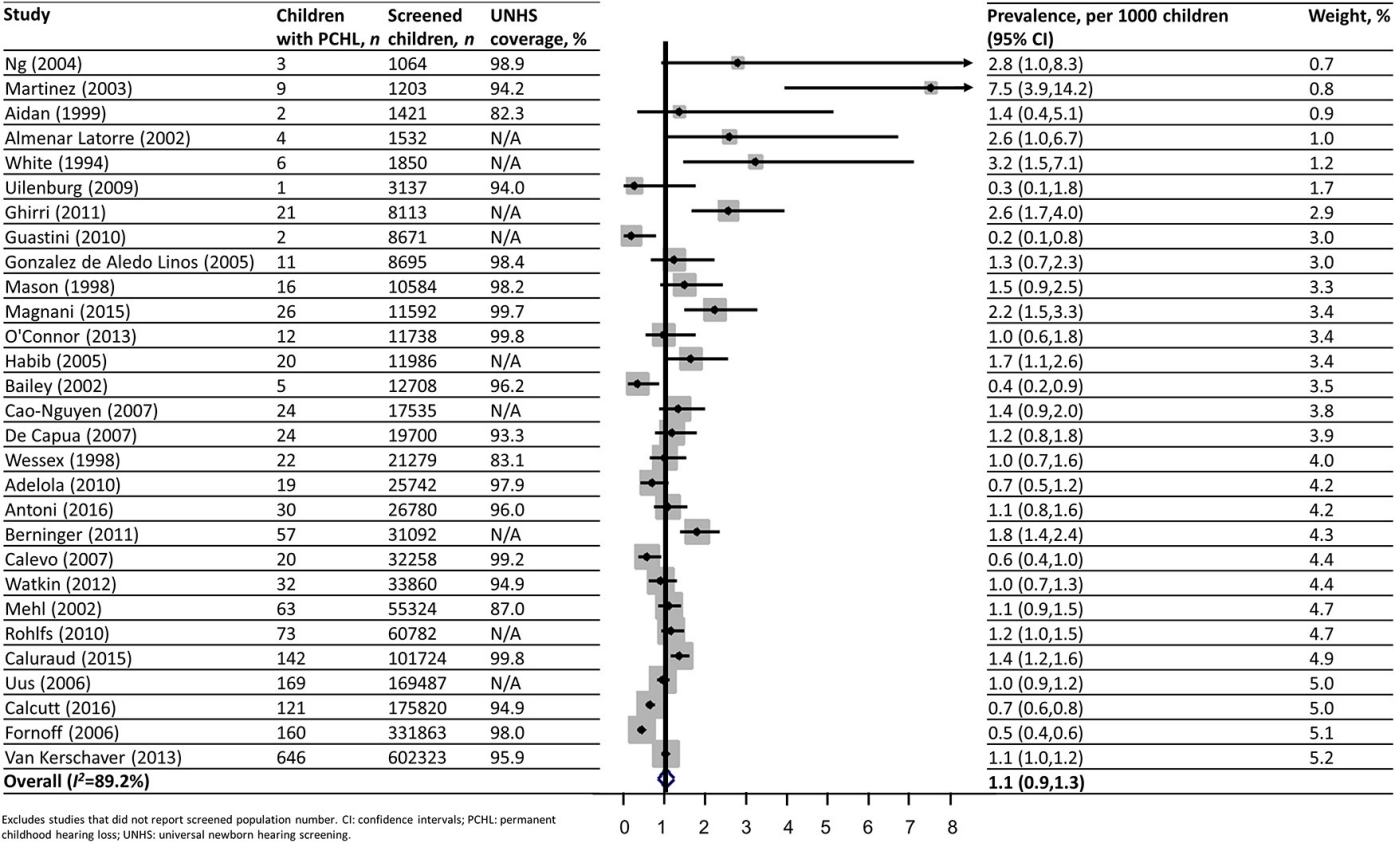
UNHS-detected PCHL prevalence in the screened population.

Pooled prevalence of UNHS-detected PCHL in the screened population was 1.1 (95% CI: 0.9, 1.3) per 1000 children (*I*^*2*^ = 89.2%) (Fig 3). Sensitivity analyses excluding studies with outlying prevalences or of low quality[48], or those with identified sample bias [22–25, 35, 38, 39, 42, 46, 49, 53, 54, 59] did not alter prevalence estimates. Similarly, prevalence estimates using the study population, rather than screened population, as the denominator did not affect estimates (prevalence: 0.9, 95% CI: 0.8, 1.1 per 1000 children).

**Fig 4:**
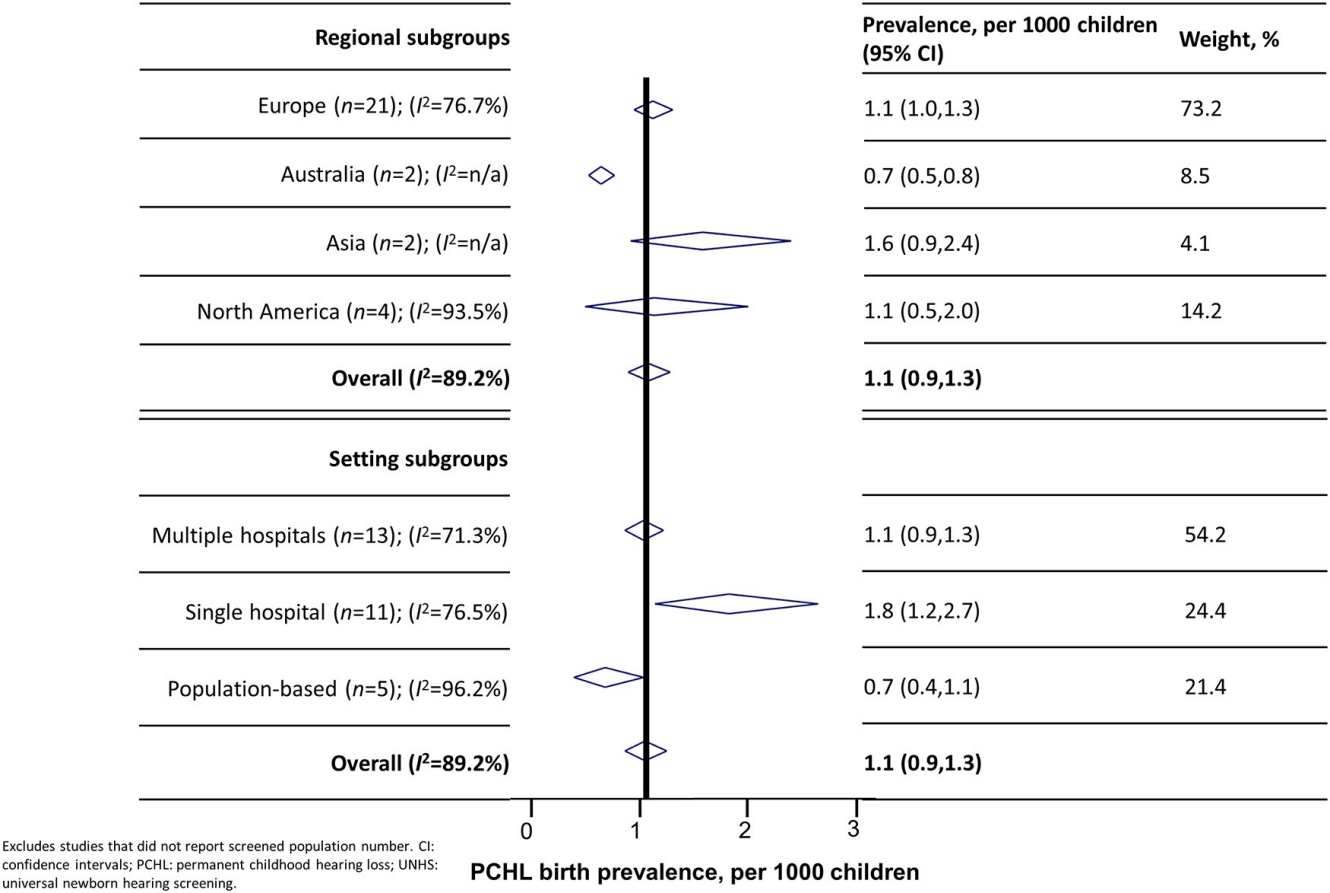
UNHS-detected PCHL prevalence in the screened population by regional and setting subgroups.

Funnel plot and Egger’s test results indicated that studies with smaller study populations produced significantly larger prevalence estimates (Egger’s test *p* = 0.02). Studies based on single hospitals had significantly smaller study populations and correspondingly higher PCHL prevalence estimates than those based in multiple hospitals or population-based (heterogeneity between settings, *p* = 0.003) (Fig 4). This association remained after exclusion of outliers [48], and was not due to significant differences in measured quality indicators (including studies with sample bias, such as exclusion of high-risk children) or study characteristics. Excluding studies with small study populations did not modify pooled prevalence estimates significantly.

Analyses of between-region heterogeneity revealed that prevalence estimates were significantly smaller for studies carried out in Australia (heterogeneity between regions, *p*<0.001) compared with other regions (Fig 4). Study characteristics or quality were similar between regions with the exception of screening protocol type (Australian studies were significantly more likely to use ABR than other protocols). Prevalence estimates did not vary significantly by screening protocol type, year started, study duration, PCHL case definition, individual quality indicators. Lack of comparability between UNHS programme protocols (for example ages of testing, number of tests, specific equipment, referral criteria) precluded investigation of associations between these factors and PCHL prevalence.

### Differences in prevalence by demographic and individual characteristics

Ethnic group of children with PCHL or of the study population was not reported in any study. Sex distribution was reported for children with PCHL fitting the review case definition in only two studies (44 and 57% female in the individual studies) [24, 55], however this was not reported for the entire study population.

**Fig 5:**
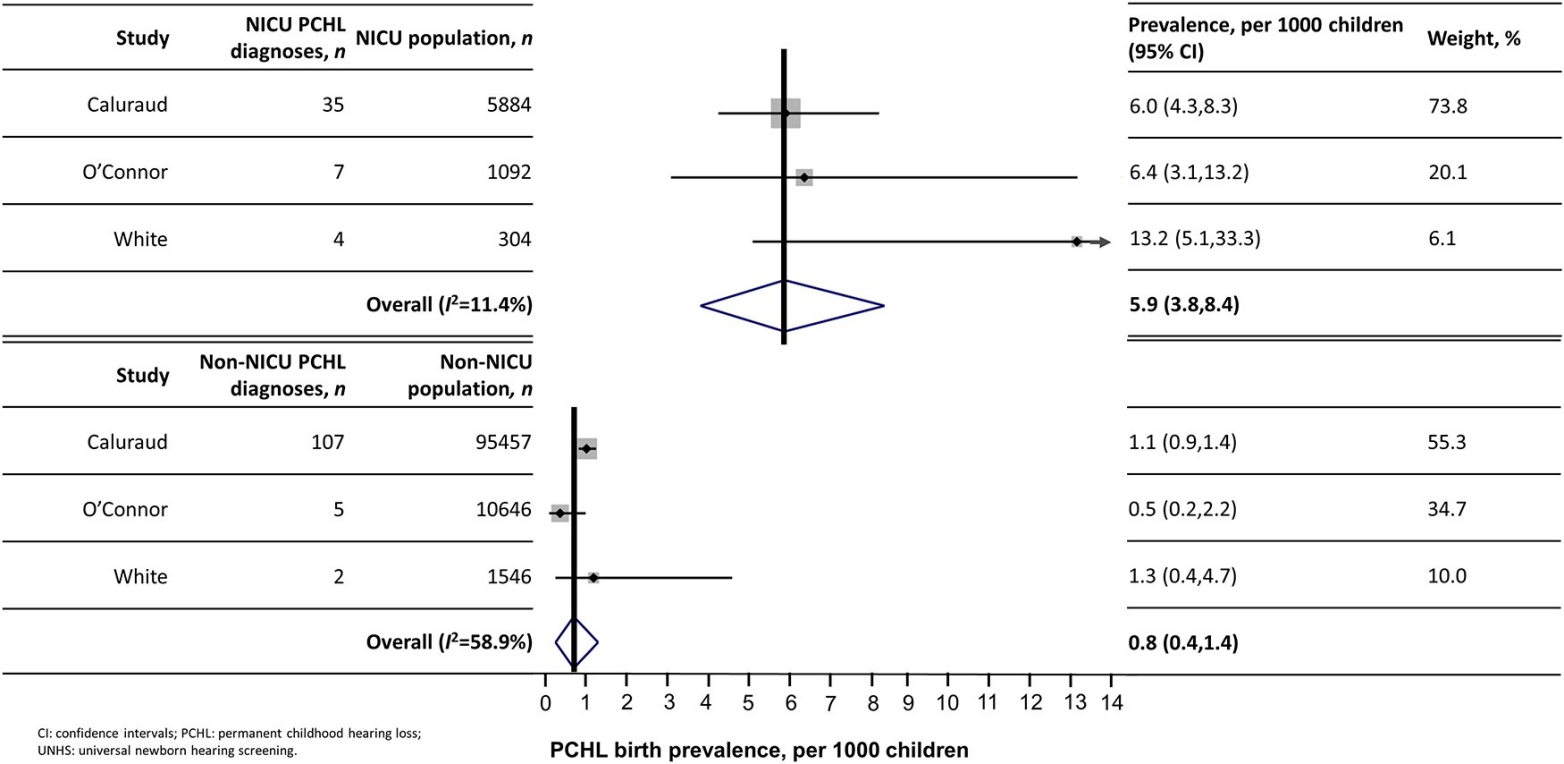
UNHS-detected PCHL prevalence in NICU versus non-NICU populations.

In three studies [30, 40, 59], information was provided on UNHS-detected PCHL prevalence by NICU admission status. Pooled PCHL prevalence was 5.9 (95% CI: 3.8, 8.4) per 1000 screened children admitted to NICU, compared with 0.8 (95% CI: 0.4, 1.4) per 1000 not admitted: a PCHL prevalence rate ratio of 6.9 (95% CI: 3.8, 12.5) (Fig 5).

### Screening programme performance

Screening programme coverage and yield is presented in Table 2 and Fig 3. In the 25 studies with available information, PPV ranged from 2% to 84% (Table 4). A median of 93% (Q1: 86%, Q3: 100%) of screen positive children attended diagnostic testing in 21 studies reporting this information.

**Table 4:** Positive predictive values for UNHS studies.

Study authors	Criteria for diagnostic referral	Referral of bilateral only (Bi), or both unilateral and bilateral (Bo), failure?	SP, *n*	TP, *n*	PPV, % 95% CI
**OAE only**					
**Uilenburg**	OAE failure	Bo	69	1	1.5 0.3–7.8
**De Capua**	OAE failure or presence of risk factors	Bo	1536	24	1.6 1.1–2.3
**González de Aledo Linos**	OAE failure or presence of risk factors	Bo	342	11	3.2 1.8–5.7
**Metzger**	OAE failure	Bi	253	15	5.9 3.6–9.6
**Habib**	OAE failure	Bo	300	20	6.7 4.4–10.1
**Ng**	OAE failure at 40 dB HL	Bo	37	3	8.1 2.8–21.3
**Aidan**	OAE failure	NS	9	2	22.2 6.3–54.7
**ABR only**					
**Mehl**	ABR failure (or OAE in few hospitals)	Bo	1283	63	4.9 3.9–6.2
**Calcutt**	ABR failure or presence of risk factors	Bo	1633	121	7.4 6.2–8.8
**Antoni**	ABR failure at 30 dB HL (modified in 2009 to only bilateral failures)	Bo	226	30	13.3 9.5–18.3
**Van Kerschaver**	ABR failure at 35 dB HL	Bo	2316	646	27.9 26.1–29.8
**OAE & ABR**					
**O'Connor**	ABR failure	Bo	525	12	2.3 1.3–4.0
**White**	ABR failure at 30 dB HL	Bo	115	6	5.2 2.4–10.9
**Wessex**	ABR failure at 35 dB HL (modified Oct 1994 to only bilateral)	Bo	392	22	5.6 3.7–8.4
**Fornoff**	Failure of last screening test (OAE or ABR)	Bo	2135	160	7.5 6.5–8.7
**Magnani**	ABR failure	Bo	241	26	10.8 7.5–15.3
**Martínez**	OAE failure or presence of risk factors	Bo	69	9	13.0 7.0–23.0
**Adelola**	ABR failure	Bo	92	19	20.7 13.6–30.0
**Bailey**	ABR failure at 35 dB HL	Bo	23	5	21.7 9.7–41.9
**Ghirri**	ABR failure	Bo	84	21	25.0 17.0–35.2
**Guastini**	ABR failure at 40 dB HL	Bo	6	2	33.3 9.7–70.0
**Rohlfs**	ABR failure at 35 dB HL	Bo	217	73	33.6 27.7–40.2
**Almenar Latorre**	ABR failure at 40 dB HL	Bo	11	4	36.4 15.2–64.6
**Calevo**	ABR failure	Bo	41	20	48.8 34.3–63.5
**Caluraud**	ABR failure at 35 dB HL	Bo	170	142	83.5 77.2–88.4

ABR: auditory brainstem responses test; NS: not stated; OAE: otoacoustic emissions test; PPV: positive predictive value; SP: screen positives; TP: true positives; UNHS: universal newborn hearing screening.

The Wessex study was deemed to have highest quality follow-up after UNHS and diagnostic testing, with active and passive follow-up to age 9 years, using multiple sources and excluding acquired, late-onset, or progressive PCHL. Sensitivity and specificity for this study were 92% (95% CI: 74%, 98%) and 98% (95% CI: 98%, 98%), respectively. NPV was 100% (95% CI: 100%, 100%). The remaining nine studies used less robust methods to follow-up screen negatives, as described earlier, however they reported similar screen performance measures to the Wessex study; overall NPV was 100% in the seven studies with available information, sensitivity 89–100% (for eight studies) and specificity 92–100% (for seven studies) (Table 5). Only 4 studies reported loss to follow-up of children in the screen negative group; this was ≤2%.

**Table 5:** Negative predictive value, sensitivity, and specificity for studies with follow-up.

Study authors	SP, *n*	TP, *n*	SN, *n*	FN, *n*	NPV, % 95% CI	Sensitivity, % 95% CI	Specificity, % 95% CI
**Almenar Latorre**	11	4	1521	0	100.0 99.9–100.0	100.0 51.0–100.0	99.5 99.6–99.8
**Berninger**	n/a	57	n/a	0	n/a	100.0 93.7–100.0	n/a
**Calcutt**	1633	121	174187	10	100.0 100.0–100.0	92.4 86.5–95.8	99.1 99.1–99.2
**Calevo**	41	20	32217	0	100.0 100.0–100.0	100.0 83.9–100.0	99.9 99.9–100.0
**Cao-Nguyen**	n/a	24	n/a	2	n/a	92.3 75.9–97.9	n/a
**De Capua**	1536	24	18164	1	100.0 100.0–100.0	96.0 80.5–99.3	92.3 91.9–92.7
**Ng**	37	3	1027	0	100.0 99.6–100.0	100.0 43.9–100.0	96.8 95.6–97.7
**O'Connor**	525	12	11213	1	100.0 100.0–100.0	92.3 66.7–98.6	95.6 95.2–96.0
**Watkin**	n/a	32	n/a	4	n/a	88.9 74.7–95.6	n/a
**Wessex**	392	22	20887	2	100.0 100.0–100.0	91.7 74.2–97.7	98.3 98.1–98.4

CI: confidence intervals; FN: false negatives; n/a: not given or could not be calculated; NPV: negative predictive value: SN: screen negatives; SP: screen positives; TP: true positive

## Discussion

### Key findings

We estimate that UNHS programmes in VHD countries identify PCHL in 1 out of every 1000 children screened. This was lower in studies carried out in Australia and higher in studies carried out in single hospitals, which included smaller study populations relative to population-based studies or those based on several hospitals. The highest prevalence was found in infants admitted to NICU, a group known to be at higher risk of PCHL, compared with those who were not. No studies reported ethnic group and only two studies reported sex, precluding estimation of sex- or ethnic-specific pooled prevalences.

Analysis of screening programme performance demonstrated good population coverage with high detection rates, however PPV varied widely across studies. Although NPV, sensitivity and specificity appeared high, these could only be estimated from studies with follow-up of screen negatives, which had marked variation in methods for, and completeness of, follow-up.

### Strengths and limitations

Strengths of this systematic review and meta-analysis include prospective publication of our protocol on PROSPERO, the systematic search strategy employed, as well as the inclusion of unpublished literature and articles in all languages, which reduced likelihood of inclusion bias. Independent article selection, data extraction, and quality assessment by multiple reviewers reduced risk of bias or error. Finally, we used robust statistical methods, including random-effects models, to calculate prevalence, screening programme performance and to examine heterogeneity.

Our searches were not restricted by language and included unpublished literature, including UNHS programme reports and evaluations, however we cannot exclude the possibility that we failed to identify relevant unpublished evidence. Our findings are generalisable to VHD countries only, reflecting the selection criteria employed, and we cannot assume they apply to other settings. Our analyses were constrained by the limited reporting of demographic and individual characteristics including on sex, ethnic group and age at diagnosis, loss to follow-up of those with screen positive results, as well as lack of reporting on follow-up (active or passive) with which to identify children diagnosed at older ages. Later PCHL diagnoses may reflect failures of screening, diagnosis, or management, or variation in natural history resulting in PCHL of progressive or later onset. Few studies employed high-quality active ascertainment of later diagnoses limiting the studies available for estimating NPV, sensitivity, and specificity. We were unable to assess bias due to selective attrition in screen performance estimates as this was rarely reported; however the consistently reported high attendance at diagnostic testing reduced the likelihood of bias in PPV estimates.

### Interpretation

Our pooled estimate of PCHL prevalence of around 1 per 1000 children is consistent with that reported from existing studies of congenital PCHL prevalence [60] prior to UNHS, suggesting that UNHS detects most newborns with early-onset PCHL. We did not detect differences in UNHS-detected PCHL prevalence by study date or target PCHL definition. There was also no association between screening protocol type and detected prevalence. This is consistent with at least one previous study comparing an OAE-only versus ABR-only protocol [61] and indicates that reported variation in referral rates by protocol type has little impact on detected prevalence [62]. Protocol type may influence programme performance and cost-effectiveness, however, further research is required to explore this. We found a higher prevalence of PCHL in studies reporting the experience of single hospitals, in comparison to those based on multiple hospitals or whole populations. The single hospital studies tended to have smaller samples than the other setting types. The difference in prevalence by settings or sample size was not clearly attributable to the measured quality indicators, including risk of sample bias, or other study characteristics. It may be that unmeasured differences in the study populations explain this finding, for instance differences in the NICU population size and characteristics.

Demographic factors, such as sex and ethnicity, which may provide insights into potential causal mechanisms, were largely not reported. This restricted exploration of variations in prevalence by these characteristics, and might have resulted in the large *I*^*2*^ value. In particular, we were unable to examine whether ethnic variation explained regional variations in prevalence. For example, Martínez *et al* suggested that the high prevalence in their study may reflect the high proportion of children of Roma ethnic origin in their study population [48]. Although there is no evidence that children of Roma ethnicity are at higher risk of PCHL, two studies have suggested other ethnic differences in hearing loss [63, 64]. The small number of studies in regions other than Europe may have reduced statistical power to detect regional differences in prevalence.

Prevalence of PCHL in babies admitted to NICU was almost seven times higher than for those not admitted. This is consistent with our previous finding based on a UK-wide cohort study whereby NICU or special care baby unit admission was associated with 6.3 (95% CI: 2.3, 17.6) times higher risk of PCHL, and neonatal illness without NICU admission with 2.6 (95% CI: 1.2, 6.0) times higher risk, than children with no neonatal illness [65]. The association between NICU and high PCHL risk may result from the underlying cause of PCHL, for example craniofacial anomalies or other syndromal pathologies, as well as exposure to ototoxic antibiotics, prolonged mechanical ventilation and asphyxia, hyperbilirubinaemia, and high noise levels in NICU [1, 4, 66, 67].

UNHS programme screening, diagnostic, and follow-up protocols varied greatly across studies. We therefore did not combine PPV estimates due to differences in screening equipment, testing protocol (including choice of stages and number of repeats), tester training, referral criteria and age at testing [68–71]. NPV, sensitivity and specificity are also affected by these factors, as well as by the strategies employed to ascertain children with later PCHL diagnoses and false negatives. PPV varied widely between studies whilst NPV, sensitivity and specificity estimates were reasonably consistent where they could be calculated. This reinforces findings from a previous review suggesting that guidelines to ensure standardised, high quality public reporting of UNHS programme performance are required [10]. Further evaluation, using adequate follow-up to detect false negatives, is justified to inform improvements in the quality and performance of screening programmes. Adequate follow-up should also involve assessing hearing threshold changes in children diagnosed with PCHL, as these can fluctuate or normalise over time [23, 24], reflecting the difficulty in conclusively diagnosing PCHL in very young children [72].

## Conclusions

We estimate that in VHD countries, audiological and other services will be required for around 1 per 1000 children with PCHL following UNHS screening, however this may vary depending on the proportion of children admitted to NICU, in whom PCHL prevalence is much greater. Future research to investigate differences in PCHL prevalence by sex and ethnicity is required, and to compare the performance of different screening protocols to identify those that are most effective. Both will depend on the quality of data collection and reporting, including attrition, and implementation of active follow-up measures to ascertain false negatives.

## Supporting information

S1 Table MOOSE checklist. (DOC)

S2 Table PRISMA checklist. (DOC)

S3 Table UNHS protocols. (DOCX)

S1 File Search strategy. (DOCX)

S2 File REDCap extraction form. (PDF)

S3 File QUADAS-2 scoring. (DOCX)
