# Supplementary material for: Prevalence of permanent childhood hearing loss detected at the universal newborn hearing screen: Systematic review and meta-analysis
Source: PLoS One. 2019 Jul 11;14(7):e0219600. doi: 10.1371/journal.pone.0219600 (PMC6622528; doi:10.1371/journal.pone.0219600)
Supplement: S3 Table — (DOCX) [file pone.0219600.s003.docx]

**S3 Table. UNHS protocols in the included studies**

| **Study** | **Protocol** | **Target population** | **Stages of protocol** |
| --- | --- | --- | --- |
| **OAE only** |  |  |  |
| **Single protocol** |  |  |  |
| Metzger | 1 | All children | OAE → OAE, ABR & diagnostic testing |
| Ng | 1 | All children | OAE, OAE, OAE → diagnostic testing (only if failed 3rd OAE) |
| Aidan | 1 | All children | OAE → OAE → diagnostic testing |
| Habib | 1 | All children - only reported on children w/o risk factors | OAE → OAE → diagnostic testing |
| Uilenburg | 1 | All children (NICU excluded) | OAE → OAE → OAE → diagnostic testing |
| **Multiple protocols** |  |  |  |
| De Capua | 1 | Children w/o risk factors | OAE → OAE → diagnostic testing |
|  | 2 | Children with risk factors | OAE & diagnostic testing |
| Gonzalez de Aledo Linos | 1 | Children w/o risk factors | OAE → OAE → diagnostic testing |
|  | 2 | Children with risk factors | OAE & diagnostic testing |
| Watkin | 1 | Children born before 2002 | OAE → OAE → diagnostic testing |
|  | 2 | Children born during 2002 | OAE → ABR → diagnostic testing |
| **ABR only** |  |  |  |
| **Single protocol** |  |  |  |
| Antoni | 1 | All children | ABR → ABR → diagnostic testing |
| Calcutt | 1 | All children | ABR → ABR → diagnostic testing |
| Mason | 1 | All children | ABR → ABR → diagnostic testing |
| NSW | 1 | All children | ABR → ABR → diagnostic testing |
| Van Kerschaver | 1 | All children | ABR → ABR → diagnostic testing |
| **Multiple protocols** |  |  |  |
| Mehl | 1 | 52/57 hospitals in 1999 | ABR then diagnostic testing - unclear number |
|  | 2 | 3/57 hospitals in 1999 | OAE then diagnostic testing - unclear number |
|  | 3 | 2/57 hospitals in 1999 | OAE & ABR then diagnostic testing - unclear number |
| **OAE and ABR** |  |  |  |
| **Single protocol** |  |  |  |
| Berninger | 1 | All children | OAE → OAE → OAE → OAE & ABR → ABR → diagnostic testing |
| Wessex trial | 1 | All children | OAE → ABR → diagnostic testing |
| **Multiple protocols** |  |  |  |
| Adelola | 1 | Well-baby nursery | OAE → OAE → ABR → diagnostic testing |
|  | 2 | NICU | OAE & ABR → diagnostic testing |
| Almenar Latorre | 1 | Children w/o risk factors | OAE → OAE → ABR → diagnostic testing |
|  | 2 | Children with risk factors | OAE & ABR → diagnostic testing |
| Bailey | 1 | Well-baby nursery | OAE → ABR → OAE +/- ABR → diagnostic testing |
|  | 2 | Long-term NICU level 2/3 | ABR → diagnostic testing - unclear number |
| Calevo | 1 | Children w/o risk factors | OAE → OAE → ABR → ABR → diagnostic testing |
|  | 2 | Children with risk factors | ABR → ABR → diagnostic testing |
| Caluraud | 1 | Children w/o risk factors | OAE → ABR → ABR → diagnostic testing |
|  | 2 | Children with risk factors | ABR → ABR → diagnostic testing |
| Cao-Nguyen | 1 | Children born 2000-2002 & infants w/o risk factors born 2002-2004 | OAE → OAE → ABR → diagnostic testing |
|  | 2 | Children with risk factors (from 2002) | OAE & ABR → diagnostic testing |
| Fornoff | 1 | Varied by hospital | OAE → OAE (+/- → OAE) → diagnostic testing |
|  | 2 | Varied by hospital | ABR → ABR (+/- → ABR) → diagnostic testing |
|  | 3 | Varied by hospital | 2-3 screening stages of OAE & ABR |
| Ghirri | 1 | Children w/o risk factors | OAE → OAE & ABR → diagnostic testing |
|  | 2 | Children with risk factors | OAE & ABR → OAE & ABR → diagnostic testing |
| Guastini | 1 | Children w/o risk factors | OAE → OAE → ABR → ABR → diagnostic testing |
|  | 2 | Children with risk factors | OAE & ABR → ABR → diagnostic testing |
| Magnani | 1 | Children w/o risk factors | OAE → OAE → ABR → diagnostic testing |
|  | 2 | Children with risk factors | OAE & ABR → diagnostic testing |
| Martinez | 1 | Children w/o risk factors | OAE → OAE → ABR & diagnostic testing |
|  | 2 | Children with risk factors | OAE, ABR & diagnostic testing |
| O'Connor | 1 | Well-baby nursery | OAE → ABR → diagnostic testing |
|  | 2 | NICU | OAE & ABR → diagnostic testing |
| Rohlfs | 1 | Well-baby nursery & NICU without risk factors | OAE → ABR → OAE → diagnostic testing |
|  | 2 | NICU with risk factors for hearing loss | ABR → ABR → ABR → diagnostic testing |
| Uus | 1 | Well-baby nursery | OAE → ABR → diagnostic testing |
|  | 2 | NICU | OAE & ABR → diagnostic testing |
| Van der Ploeg | 1 | Well-baby nursery hospitalised for <3 weeks | OAE → OAE → ABR → diagnostic testing |
|  | 2 | Well-baby nursery hospitalised for >3 weeks | ABR → ABR → diagnostic testing |
| White | 1 | Random sample | OAE & ABR → OAE and/or ABR → diagnostic testing |
|  | 2 | Random sample | OAE → OAE and/or ABR → diagnostic testing |

ABR: auditory brainstem response testing; NICU: neonatal intensive care unit; OAE: otoacoustic emissions; w/o: without. ‘→’ refers to a testing stage, only infants failing at the previous testing stage are referred to the following stage.
