# Supplementary material for: Prevalence of permanent childhood hearing loss detected at the universal newborn hearing screen: Systematic review and meta-analysis
Source: PLoS One. 2019 Jul 11;14(7):e0219600. doi: 10.1371/journal.pone.0219600 (PMC6622528; doi:10.1371/journal.pone.0219600)
Supplement: S1 File — (DOCX) [file pone.0219600.s004.docx]

***S1 File: Search strategy***

**Pubmed**

| **#** | **Searches** | **Results** |
| --- | --- | --- |
| 1 | ("hearing loss"[MeSH Terms] OR ("hearing"[All Fields] AND "loss"[All Fields]) OR "hearing loss"[All Fields]) | 76478 |
| 2 | Hearing impair* | 14147 |
| 3 | "deaf"[All Fields] OR ("deafness"[MeSH Terms] OR "deafness"[All Fields]) | 40463 |
| 4 | 1 OR 2 OR 3 | 88834 |
| 5 | "epidemiology"[Subheading] OR "epidemiology"[tiab] OR "incidence"[tiab] OR "incidence"[MeSH Terms] OR "prevalence"[tiab] OR "prevalence"[MeSH Terms] | 2459807 |
| 6 | 4 AND 5 | 10800 |
| 7 | child*[tiab] or infant*[tiab] or adolescen*[tiab] or newborn*[tiab] or neonat*[tiab] | 1771311 |
| 8 | 6 AND 7 | 3979 |

**OvidSP(medline)**

| **#** | **Searches** | **Results** |
| --- | --- | --- |
| 1 | exp Hearing Loss/ | 66394 |
| 2 | exp Deafness/ | 27871 |
| 3 | 1 or 2 | 66394 |
| 4 | (hearing and loss).ti,ab,kw. | 42643 |
| 5 | (hearing and impair*).ti,ab,kw. | 17343 |
| 6 | deaf*.ti,ab,kw. | 37412 |
| 7 | 1 or 4 or 5 or 6 | 101912 |
| 8 | incidence.mp. [mp=title, abstract, original title, name of substance word, subject heading word, keyword heading word, protocol supplementary concept word, rare disease supplementary concept word, unique identifier] | 781259 |
| 9 | incidence/ or prevalence/ | 478014 |
| 10 | (epidemiology or incidence or prevalence).mp. [mp=title, abstract, original title, name of substance word, subject heading word, keyword heading word, protocol supplementary concept word, rare disease supplementary concept word, unique identifier] | 1475521 |
| 11 | (epidemiology or incidence or prevalence).ti,ab,kw. | 1271290 |
| 12 | 9 or 11 | 1425533 |
| 13 | exp adolescent/ or exp child/ or child, preschool/ or infant/ or infant, newborn/ | 3531178 |
| 14 | (child* or infant* or adolescen* or newborn* or infant* or neonat*).ti,ab,kw. | 1971885 |
| 15 | 14 | 1971885 |
| 16 | 7 and 12 and 15 | 2893 |

**EMBASE**

| **#** | **Searches** | **Results** |
| --- | --- | --- |
| 1 | exp hearing impairment/ | 83913 |
| 2 | (hearing and loss).mp. | 58073 |
| 3 | (hearing and impair*).mp. | 64121 |
| 4 | deaf*.mp. | 58412 |
| 5 | 1 or 2 or 3 or 4 | 124718 |
| 6 | incidence/ or prevalence/ | 865444 |
| 7 | (epidemiology or incidence or prevalence).ti,ab,kw. | 1555994 |
| 8 | 6 or 7 | 1749778 |
| 9 | (child* or infant* or adolescen* or newborn* or infant* or neonat*).ti,ab,kw. | 2100180 |
| 10 | 9 | 2100180 |
| 11 | 5 and 8 and 10 | 4019 |

**CINAHL**

| **Search number** | **Searches** | **Results** |
| --- | --- | --- |
| 1 | (MH "Hearing Loss, Partial+") OR (MH "Deafness+") OR (MH "Hearing Loss, Sensorineural+") | 12,422 |
| 2 | TX hearing AND TX loss | 15,100 |
| 3 | TX hearing AND TX impair* | 8,375 |
| 4 | TX deaf* | 13,748 |
| 5 | Search 1 OR 2 OR 3 OR 4 | 30,968 |
| 6 | (MH "Incidence") OR (MH "Prevalence") | 98,289 |
| 7 | TI incidence OR AB incidence OR TI prevalence OR AB prevalence OR TI epidemiology OR AB epidemiology | 191,101 |
| 8 | Search 6 OR 7 | 240,485 |
| 9 | Search 5 and 8 | 1,800 |
| 10 | TI adolescen* OR AB adolescen* OR TI child* OR AB child* OR TI infant* OR AB infant* OR TI newborn* OR AB newborn* OR TI neonat* OR AB neonat* | 427.286 |
| 11 | Search 9 AND 10 | 613 |

**Cochrane library**

((deaf*) or ("hearing" and "impair*") or hearing loss) and (incidence or prevalence) and (child* or infant* or neonat* or adolescen* or newborn*)

**Google Scholar**

((deaf*) or ("hearing" and "impair*") or hearing loss) and (incidence or prevalence) and (child* or infant* or neonat* or adolescen* or newborn*)
