# Supplementary material for: Prevalence of permanent childhood hearing loss detected at the universal newborn hearing screen: Systematic review and meta-analysis
Source: PLoS One. 2019 Jul 11;14(7):e0219600. doi: 10.1371/journal.pone.0219600 (PMC6622528; doi:10.1371/journal.pone.0219600)
Supplement: S2 File — (PDF) [file pone.0219600.s005.pdf]

Data extraction

Record ID ______________________

Article details

Paper number ______________________
(Check excel sheet)

Study number ______________________
(Check excel sheet)

Reviewer ◯ EB ◯ RK ◯ CD

Title of paper ______________________

First author ______________________
(format: Last name, First name.)

Year published ______________________

Is this paper a systematic review of UNHS studies - if yes, tick yes and stop extraction
◯ Yes
◯ No

Country ______________________

Study eligibility.

For all following sections, please extract data only for children undergoing UNHS, i.e. if multiple samples are reported in the article, with only one sample undergoing UNHS, only the relevant sample should be considered in extraction

Check against exclusion criteria - if any box except 'none of the above' is ticked, stop and email EB re: exclusion

- ☐ No English abstract
- ☐ Non-systematic review, comment piece, letter, editorial, case report less than 15 participants
- ☐ Not in a very highly-developed country
- ☐ UNHS not in place/being tested (defined as OAE and/or ABR testing of all infants, along with diagnostic testing, starting within 6 months of birth)
- ☐ Cannot calculate total number of children with confirmed non-transient bilateral hearing impairment more than or equal to 26 dB HL detected at UNHS
- ☐ Does not report total number of children undergoing screening
- ☐ Reporting on a selective sample (unless all non-NICU/at risk)
- ☐ All patients aged >1 year at start of study
- ☐ Minimum severity more than or equal to 61 dB HL
- ☐ Numerator bias present which can't be accounted for
- ☐ None of the above - continue

Any further notes to record for this section

____
(-1 if no notes)

Study design

Are the start and finish dates (including day, month and year) of data collection specified?
- ◯ Yes
- ◯ No or unclear

Date started data collection
____
(Format DD-MM-YY. If no day/month provided, put in '1' for relevant missing value)

Date finished data collection
____
(Format DD-MM-YY. If no day/month provided, put in '1' for relevant missing value)

If no date provided for start/finish - give any relevant information on dates/duration of data collection here
____

Setting
- ◯ Population-based including infants not born in hospitals
- ◯ Hospital based - multiple centres
- ◯ Hospital based - single centre
- ◯ Other
- ◯ NS

If hospital based, are hospital births likely to be representative of population?
- ◯ Yes
- ◯ No
- ◯ Unclear

Specify details if setting is 'other'
____

Study design type
- ◯ Pilot study, intervention study, UNHS trial or study testing feasibility of UNHS
- ◯ Cohort study
- ◯ Initial cross-sectional study with follow-up
- ◯ Cross-sectional study without follow-up
- ◯ Case series
- ◯ Other
- ◯ NS

(Classify all stages of UNHS, including diagnostics, as being cross-sectional)

If 'other' study design, describe design
____

Sampling procedure
- ◯ Approached all children in the population/hospitals included in the study
- ◯ Approached selected sample of the population (e.g. excluded NICU infants)
- ◯ Random sample
- ◯ Other
- ◯ NS

If the study used a selective sample, random sample or other sampling procedure in above question, describe how the sample were selected

____
(Highlight any concerns over representativeness)

Any further notes to record for this section

____

Sample participants

Are NICU/at-risk infants included in the study?

- ◯ No
- ◯ Yes
- ◯ Unclear

How are 'at-risk' infants defined?

JCIH 2007: Caregiver concern regarding hearing, speech, language, or developmental delay, Family history, NICU >5 days, ECMO, assisted ventilation, ototoxic medications, hyperbilirubinemia requiring exchange transfusion, In-utero infections, Craniofacial anomalies, Syndromes associated with hearing loss, Neurodegenerative disorders, sensory motor neuropathies, Postnatal infections associated with SNHL (including meningitis), Head trauma, Chemotherapy

JCIH 1994: Family history, in utero infection, craniofacial anomalies, low birth weight, hyperbilirubinaemia, ototoxic medications, meningitis, Apgar 0-4 at 1 minute or 0-6 at 5 minutes, Mechanical ventilation lasting 5 days or more, physical findings indicative of a syndrome known to include SNHL or CHL, parent/caregiver concern, head trauma, recurrent otitis media with effusion, neurodegenerative disorders

- ☐ NS or unclear
- ☐ Presence of any JCIH 2007 position statement risk factors
- ☐ Presence of any JCIH 1994 position statement risk factors
- ☐ NICU/equivalent admission
- ☐ Exposure to ototoxic mediations
- ☐ Family history of hearing impairment
- ☐ Craniofacial abnormalities
- ☐ Low birth weight
- ☐ Low Apgar scores
- ☐ Stigmata or other indication of a syndrome associated with HI
- ☐ Prenatal or perinatal infection
- ☐ Hyperbilirubinaemia
- ☐ Mechanical ventilation
- ☐ Other

If 'other' criteria were used, what were they?

____

Any further notes to record relating to eligibility criteria

____
(Enter -1 if none)

Study case definition - the review case definition is 'hearing impairment >=26 dB HL, bilateral and non-transient'

Is the total number of hearing impairments that are >=26 dB HL, bilateral and non-transient reported in the article?

- ◯ Yes
- ◯ No

(If no, stop and discuss)

What was the planned or actual minimum severity of hearing impairment in dB HL included in the article (if only in dB SPL, mark as -1)?
Note:
1. This should not refer to the filter threshold/signal to noise ratio.
2. If 'planned' severity is unclear, check the actual minimum reported severity is and report this.

____
(State -1 if NS)

Is this the planned or actual minimum severity?

____
(State -1 if NS. 'Actual' refers to the minimum reported severity in the results section.)

Is the planned/actual minimum severity of hearing impairment < 26 dB HL?

- ◯ Unclear
- ◯ Yes, and unclear how many were over 26 dB HL and fitted the review case definition
- ◯ Yes, but can clearly separate hearing impairment fitting the review case definition (i.e. at least 26 dB HL, bilateral and non-transient)
- ◯ No

If the minimum is < 26 dB HL, what is the minimum severity of hearing impairment that fit the case definition of the review (>=26 dB HL, bilateral, non-transient)?

____
(Specify in dB HL)

If the minimum severity is unclear, or if it is unclear how many are >26 dB HL, discuss whether paper should be excluded

____

What are the included anatomies of hearing impairment that fit the case definition of the review?

- ◯ SNHL
- ◯ CHL
- ◯ Mixed
- ◯ All
- ◯ SNHL & mixed
- ◯ NS
- ◯ Other

If 'other' anatomy types, specify types included

____

Are both unilateral and bilateral impairment included in the reported hearing impairments?

- ◯ Bilateral only
- ◯ Unilateral & bilateral
- ◯ Unilateral only; exclude
- ◯ NS or unclear

If unilateral and bilateral impairments are included, is the number of bilateral cases meeting the case definition reported clearly?

- ◯ Unclear
- ◯ Yes
- ◯ No

(If unsure, stop and discuss)

Are transient impairments included in article? If so, are the number of non-transient impairments fitting the review case definition clear?

- ◯ Transient impairments not reported
- ◯ Transient impairment reported, but clearly separated from non-transient impairment fitting the review case definition
- ◯ Transient impairment included, not clearly separated from non-transient impairment fitting the review case definition - stop and discuss exclusion

Any further notes to record for this section

____

This section should be filled in on either the only protocol used, or in cases with multiple protocols, the protocol used for the majority of children or for children not at high risk of hearing impairment

By what age were infants usually first screened? ______________________ (Specify age in days or -1=NS)

What stages of testing were used?
- ◯ OAE & diagnostic investigation
- ◯ ABR & diagnostic investigation
- ◯ OAE, ABR & diagnostic investigation - only receive ABR if failed OAE
- ◯ OAE, ABR & diagnostic investigation - must receive OAE & ABR
- ◯ Other - consider exclusion if no OAE/ABR & diagnostics
- ◯ NS

If 'other' stages of testing used, please describe ______________________

Usual age by which diagnostic testing stage completed ______________________ (Specify age in months or -1=NS)

What was the minimum number of tests infants could undergo excluding the diagnostic stages? ______________________ (Specify number or -1 if unclear)

What was the maximum number of tests infants could undergo excluding the diagnostic stages? ______________________ (Specify number or -1 if unclear)

Were both unilateral and bilateral fails referred to diagnostic investigation?
- ◯ Yes
- ◯ No, bilateral only
- ◯ No, unilateral only
- ◯ Unclear

What was the minimum severity, in dB HL, that was considered grounds for referral to diagnostics? ______________________ (State number or -1 if unclear)

Please answer this section for the second UNHS protocol in place

Is there a second protocol in place?
- ☐ No
- ☐ Unclear
- ☐ Yes - separate for NICU infants
- ☐ Yes - separate if had risk factors (other than just NICU admission)
- ☐ Yes - separated by another criteria

What criteria were used? ______________________

By what age were infants usually first screened? ______________________ (Specify approximate age in days or -1=NS)

What stages of testing were used?

◯ OAE & diagnostic investigation
◯ ABR & diagnostic investigation
◯ OAE, ABR & diagnostic investigation - only receive ABR if failed OAE
◯ OAE, ABR & diagnostic investigation - must receive OAE & ABR
◯ Other - consider exclusion
◯ NS

If 'other' stages of testing were used, please describe

____

Usual age by which diagnostic testing stage completed

____
(Specify age in weeks or -1=NS)

What was the minimum number of tests infants could undergo excluding the diagnostic stages?

____
(Specify number or -1 if unclear)

What was the maximum number of tests infants could undergo excluding the diagnostic stages?

____
(Specify number or -1 if unclear)

Were both unilateral and bilateral fails referred to diagnostic investigation?

◯ Yes
◯ No, bilateral only
◯ No, unilateral only
◯ Unclear

What was the minimum severity, in dB HL, that was considered grounds for referral to diagnostics?

____
(State number or -1 if unclear)

Please answer this section for the third UNHS protocol in place (if relevant)

Is there a third protocol in place?

☐ No
☐ Unclear
☐ Yes - separate for NICU infants
☐ Yes - separate if had risk factors (other than just NICU admission)
☐ Yes - separated by another criteria

What criteria were used?

____

By what age were infants usually first screened?

____
(Specify approximate age in days or -1=NS)

What stages of testing were used?

◯ OAE & diagnostic investigation
◯ ABR & diagnostic investigation
◯ OAE, ABR & diagnostic investigation - only receive ABR if failed OAE
◯ OAE, ABR & diagnostic investigation - must receive OAE & ABR
◯ Other - consider exclusion
◯ NS

If 'other' stages of testing were used, please describe

____

Usual age by which diagnostic testing stage completed

____
(Specify age in weeks or -1=NS)

What was the minimum number of tests infants could undergo excluding the diagnostic stages?

____
(Specify number or -1 if unclear)

What was the maximum number of tests infants could undergo excluding the diagnostic stages?

____
(Specify number or -1 if unclear)

Were both unilateral and bilateral fails referred to diagnostic investigation?

◯ Yes
◯ No
◯ Unclear

What was the minimum severity, in dB HL, that was considered grounds for referral to diagnostics?

____
(State number or -1 if unclear)

Were any more UNHS protocols in place?

◯ Yes
◯ No

Describe any further UNHS protocols

____

Any further notes to record on any protocols

____

Follow-up

Is information given on planned/actual length of follow-up from birth in the study?

☐ No or unclear
☐ Yes - the minimum and/or maximum lengths of follow-up are specified
☐ Yes - the median follow-up and/or IQR are specified
☐ Yes - the mean follow-up and/or standard deviation are specified
☐ Other information on follow-up given
(This refers to all follow-up within UNHS or after UNHS has completed)

If 'other information', what was this?

____

What is the minimum length of follow-up?

____
(Report in months or -1 if not specified)

What is the maximum length of follow-up?

____
(Report in months or -1 if not specified)

What is the median length of follow-up?

____
(Report in months or -1 if not specified)

What is the inter-quartile range of follow-up?

____
(Report in months or -1 if not specified)

What is the mean length of follow-up?

____
(Report in months or -1 if not specified)

What is the standard deviation of follow-up?

____
(Report in months or -1 if not specified)

Does the article report on follow-up after UNHS is completed?

- ◯ Only reports on UNHS results
- ◯ Includes follow-up after UNHS stages to less than 1 year of age
- ◯ Includes follow-up after UNHS stages to equal or more than 1 year of age
- ◯ Includes follow-up after UNHS stages though unclear length

Is the number of additional diagnoses (separate to UNHS diagnoses) fitting the review case definition and made within the first year of life reported?

- ◯ Yes
- ◯ No

What methods were used to identify cases after UNHS?

- ◯ Reported to register
- ◯ Active surveillance of clinicians
- ◯ Regular testing during follow-up
- ◯ Other
- ◯ NS

Describe any details given if 'other' or 'NS' ticked for method of identifying cases after UNHS

____

Any further notes to record for this section

____

Results

Are results reported for:
Note 1: Where protocols were not separated by presence of risk factors, use the 'at-risk' category for the protocol used on the smallest number of infants and the 'not-at-risk' category for the protocol used on the largest number of infants.

Note 2: If babies at risk were excluded from the study, or if there is only one protocol, just fill in the 'all babies together' section.

- ☐ All babies together
- ☐ For at-risk separately
- ☐ For not-at risk separately
- ☐ For any further protocols

(Check all that apply, even if only some information is available for a category e.g. just total number considered for all babies)

Any notes to report on the results section?

____
(-1 if none)

Fill in this section with results at UNHS testing for all babies

For the denominator in the prevalence calculation (i.e. total number of children screened), do the results for all babies report:

- ☐ The number of children considered for inclusion, whether or not they received any screening (study population)?
- ☐ The number of children receiving at least one screening test, including those that did not complete screening?
- ☐ The number of children completing screening (i.e. followed-up until normal/abnormal hearing was confirmed)?
- ☐ The number of children 'screened' where it is unclear if this includes children that dropped out/were lost to follow-up
- ☐ Unclear or does not fit into above categories

(Select all applicable)

If 'unclear' option is ticked above, describe any details given on the numbers offered/receiving screening & whether definitely eligible for inclusion

____

Report study population - total number considered for inclusion in the study including non-screened

____
(Specify number or -1=NS)

Total number reported to not receive any screening in the study

____
(Specify number or -1=NS)

Report the recruitment/population coverage (as a % of study population) of UNHS

____
(Report as a percentage (i.e. 50), or -1 if NS)

Report the reasons given for non-participation in UNHS (and numbers if available).

____
(Only include children not-participating at the first stage of UNHS. Fill in with -1 if not reported)

Report total number that received at least one screening test (even with later drop-out)

____
(Specify number or -1=NS)

How many children were lost to follow up and/or did not receive confirmation of hearing status at UNHS after receiving at least one test?
Just report total number - give reasons in next question.

N.b. Do not include number not receiving any hearing test

____
(Specify number or -1=NS)

Report reasons & numbers for those lost to follow-up and/or not receiving confirmation of hearing loss

____
(-1 if not reported)

How many children were lost to follow up before referral to diagnostics?

____
(Specify number or -1=NS)

How many children were lost to follow up at the diagnostic stage?

____
(Specify number or -1=NS)

How many children finished the screening, including diagnostic stages (i.e. did not drop out/get lost to follow-up)?

____
(Specify number or -1=NS)

How many children were referred after the first stage of the screening test? | ______________________ (-1 if NS)

Report total number receiving screening (where it is unspecified if children lost to follow up are included) | ______________________ (Specify number or -1=NS)

How many children were screen negative and not referred for diagnostic investigation? | ______________________ (-1 if NS)

How many children were referred for diagnostic investigation? | ______________________ (-1 if NS)

Total number of confirmed hearing impairments detected at UNHS that fit case definition of this review | ______________________ (Specify number or -1=NS)

Total number confirmed to have hearing impairment at UNHS that did not fit review case definition | ______________________ (Specify number or -1=NS)

Specify reasons for not meeting case definition and numbers

______________________ (Give numbers & reason)

Total number confirmed to have normal hearing at diagnostic investigation | ______________________ (Specify number or -1=NS)

What further information is given on screen negatives after UNHS?

- ☐ None - no further confirmation of normal/abnormal hearing
- ☐ Report of number later confirmed to have normal hearing
- ☐ Report of number later confirmed to have hearing impairment

Report number later confirmed to have normal hearing ______________________

Report number later confirmed to have hearing impairment fitting the review case definition ______________________

Report number later confirmed to have hearing impairment not fitting the review case definition ______________________

Describe reasons provided for changes in hearing status after UNHS

______________________ (report -1 if not specified)

Is further information is given on changes in hearing status for screen positives after UNHS?

- ☐ No further confirmation of normal/abnormal hearing
- ☐ Report of number with normal hearing diagnosed later confirmed to have hearing impairment
- ☐ Report of number with hearing impairment later confirmed to have normal hearing

Report number later confirmed to have normal hearing ______________________

Report number later confirmed to have hearing impairment fitting the review case definition ______________________

Report number later confirmed to have hearing impairment not fitting the review case definition ______________________

Describe reasons provided for changes in hearing status after UNHS

____
(report -1 if not specified)

Any notes on this section

____
(-1 if none)

Fill in this section using numbers reported for children not at high risk of hearing impairment (i.e. not in NICU, no risk factors, and/or not requiring high risk protocol)

For the denominator in the prevalence calculation (i.e. total number of children screened), for children in the not at risk protocol does the article report:

- ☐ The number of children considered for inclusion, whether or not they received any screening (study population)?
- ☐ The number of children receiving at least one screening test, including those that did not complete screening?
- ☐ The number of children completing screening (i.e. followed-up until normal/abnormal hearing was confirmed)?
- ☐ The number of children 'screened' where it is unclear if this includes children that dropped out/were lost to follow-up
- ☐ Unclear or does not fit into above categories

(Select all applicable)

If 'unclear' ticked above, please describe any details given on the numbers offered/receiving screening & whether definitely eligible for inclusion

____

Report total number considered for inclusion (including non-screened)

____
(Specify number or -1=NS)

Report the recruitment/population coverage (as a % of study population) of UNHS

____
(Report as a percentage (i.e. 50), or -1 if NS)

Total number reported to not receive any screening?

____
(Specify number or -1=NS)

Report the reasons given for non-participation in UNHS (and numbers if available).

____
(Only include children not participating at first stage. Fill in with -1 if not reported)

Report total number that received at least one screening test (even with later drop-out)

____
(Specify number or -1=NS)

How many children were lost to follow up and/or did not receive confirmation of hearing status at UNHS after receiving at least one test?
Just report total number - give reasons in next question.

N.b. Do not include number not receiving any hearing test

____
(Specify number or -1=NS)

Report reasons given for children that did not receive confirmation of hearing loss & numbers in each category	______________________ (-1 if not reported)
How many children were lost to follow up before diagnostic investigation?	______________________ (Specify number or -1=NS)
How many children were lost to follow up at diagnostic investigation?	______________________ (Specify number or -1=NS)
How many children finished the screening, including diagnostic stages (i.e. did not drop out/get lost to follow-up)?	______________________ (Specify number or -1=NS)
Report total number receiving screening (where it is unspecified if children lost to follow up are included) in this protocol	______________________ (Specify number or -1=NS)
How many children were referred after the first stage of the screening test?	______________________ (-1 if NS)
How many children were screen negative and not referred for diagnostic investigation?	______________________ (-1 if NS)
How many children were screen positive and referred for diagnostic investigation?	______________________ (-1 if NS)
Total number of confirmed hearing impairments at UNHS that fit the review case definition	______________________ (Specify number or -1=NS)
Total number confirmed to have normal hearing at diagnostic investigation	______________________ (Specify number or -1=NS)
Total number confirmed to have hearing impairment that did not fit review case definition by end of the diagnostic stages	______________________ (Specify number or -1=NS)
Specify reasons for not meeting case definition and numbers	______________________ (Give numbers & reason)
What further information is given on screen negatives after UNHS?	☐ None - no further confirmation of normal/abnormal hearing<br>☐ Report of number later confirmed to have normal hearing<br>☐ Report of number later confirmed to have hearing impairment
Report number later confirmed to have normal hearing	______________________
Report number later confirmed to have hearing impairment fitting the review case definition	______________________
Report number later confirmed to have hearing impairment not fitting the review case definition	______________________
Describe reasons provided for changes in hearing status after UNHS	______________________ (report -1 if not specified)

What further information is given on changes in hearing status for screen positives after UNHS?

☐ None - no further confirmation of normal/abnormal hearing
☐ Report of number with normal hearing diagnosed later confirmed to have hearing impairment
☐ Report of number with hearing impairment later confirmed to have normal hearing

Report number later confirmed to have normal hearing ________________________________

Report number later confirmed to have hearing impairment fitting the review case definition ________________________________

Report number later confirmed to have hearing impairment not fitting the review case definition ________________________________

Describe reasons provided for changes in hearing status after UNHS

____
(report -1 if not specified)

Any notes for this section

____
(-1 f none)

Fill in this section for reported numbers in the at-risk/minority protocol

For the denominator in the prevalence calculation (i.e. total number of children screened), for children in the at risk protocol does the article report:

☐ The number of children considered for inclusion, whether or not they received any screening (study population)?
☐ The number of children receiving at least one screening test, including those that did not complete screening?
☐ The number of children completing screening (i.e. followed-up until normal/abnormal hearing was confirmed)?
☐ The number of children 'screened' where it is unclear if this includes children that dropped out/were lost to follow-up
☐ Unclear or does not fit into above categories
(Select all applicable)

If 'unclear' option ticked, please describe any details given on the numbers offered/receiving screening & whether definitely eligible for inclusion

____

Report total number considered for inclusion (including non-screened) in this protocol

____
(Specify number or -1=NS)

Report the recruitment/population coverage (as a % of study population) of UNHS

____
(Report as a percentage (i.e. 50), or -1 if NS)

Total number reported to not receive any screening in this protocol

____
(Specify number or -1=NS)

Report the reasons given for non-participation in UNHS (and numbers if available).

____
(Only include children not participating at the first stage. Fill in with -1 if not reported)

Report total number that received at least one screening test (even with later drop-out) in this protocol | ________________ (Specify number or -1=NS)

How many children were lost to follow up and/or did not receive confirmation of hearing status at UNHS after receiving at least one test?
Just report total number - give reasons in next question.

N.b. Do not include number not receiving any hearing test | ________________ (Specify number or -1=NS)

Report reasons given for children that did not receive confirmation of hearing loss & numbers in each category | ________________ (-1 if not reported)

How many children were lost to follow up before diagnostic investigation? | ________________ (Specify number or -1=NS)

How many children were lost to follow up at diagnostic investigation? | ________________ (Specify number or -1=NS)

Report total number receiving screening (where it is unspecified if children lost to follow up are included) in the at-risk protocol | ________________ (Specify number or -1=NS)

How many children finished the screening, including diagnostic stages (i.e. did not drop out/get lost to follow-up)? | ________________ (Specify number or -1=NS)

How many children were referred after the first stage of the screening test? | ________________ (-1 if NS)

How many children were screen negative and not referred for diagnostic investigation? | ________________ (-1 if NS)

How many children were referred for diagnostic investigation? | ________________ (-1 if NS)

Total number of confirmed hearing impairments that fit case definition of this review in this protocol | ________________ (Specify number or -1=NS)

Total number confirmed to have normal hearing at diagnostic investigation | ________________ (Specify number or -1=NS)

Total number confirmed to have hearing impairment that did not fit review case definition by end of UNHS | ________________ (Specify number or -1=NS)

Specify reasons for not meeting case definition and numbers in this protocol | ________________ (Give numbers & reason)

What further information is given on screen negatives after UNHS?

- ☐ None - no further confirmation of normal/abnormal hearing
- ☐ Report of number later confirmed to have normal hearing
- ☐ Report of number later confirmed to have hearing impairment

Report number later confirmed to have normal hearing | ________________

Report number later confirmed to have hearing impairment fitting the review case definition

____

Report number later confirmed to have hearing impairment not fitting the review case definition

____

Describe reasons provided for changes in hearing status after UNHS

____
(report -1 if not specified)

What further information is given on changes in hearing status for screen positives after UNHS?

- ☐ None - no further confirmation of normal/abnormal hearing
- ☐ Report of number with normal hearing diagnosed later confirmed to have hearing impairment
- ☐ Report of number with hearing impairment later confirmed to have normal hearing

Report number later confirmed to have normal hearing

____

Report number later confirmed to have hearing impairment fitting the review case definition

____

Report number later confirmed to have hearing impairment not fitting the review case definition

____

Describe reasons provided for changes in hearing status after UNHS

____
(report -1 if not specified)

Any notes for this section

____
(-1 if none)

Results for further protocols

For the denominator in the prevalence calculation (i.e. total number of children screened), for children in the at risk protocol does the article report:

- ☐ The number of children considered for inclusion, whether or not they received any screening (study population)?
- ☐ The number of children receiving at least one screening test, including those that did not complete screening?
- ☐ The number of children completing screening (i.e. followed-up until normal/abnormal hearing was confirmed)?
- ☐ The number of children 'screened' where it is unclear if this includes children that dropped out/were lost to follow-up
- ☐ Unclear or does not fit into above categories

(Select all applicable)

If 'unclear' option ticked, please describe any details given on the numbers offered/receiving screening & whether definitely eligible for inclusion

____

Report total number considered for inclusion (including non-screened) in this protocol

____
(Specify number or -1=NS)

Report the recruitment/population coverage (as a % of study population) of UNHS

____
(Report as a percentage (i.e. 50), or -1 if NS)

Total number reported to not receive any screening in this protocol?

____
(Specify number or -1=NS)

Report the reasons given for non-participation in UNHS (and numbers if available)

____
(Fill in with -1 if not reported)

Report total number that received at least one screening test (even with later drop-out) in this protocol

____
(Specify number or -1=NS)

How many children were lost to follow up and/or did not receive confirmation of hearing status after receiving at least one test?
Just report total number - give reasons in next question.

N.b. Do not include number not receiving any hearing test

____
(Specify number or -1=NS)

Report reasons given for children that did not receive confirmation of hearing loss & numbers in each category

____
(-1 if not reported)

How many children were lost to follow up before diagnostic investigation?

____
(Specify number or -1=NS)

How many children were lost to follow up at diagnostic investigation stage?

____
(Specify number or -1=NS)

Report total number receiving screening (where it is unspecified if children lost to follow up are included) in this protocol

____
(Specify number or -1=NS)

How many children finished the screening, including diagnostic stages (i.e. did not drop out/get lost to follow-up)?

____
(Specify number or -1=NS)

How many children were referred after the first stage of the screening test?

____
(-1 if NS)

How many children were screen negative and not referred for diagnostic investigation?

____
(-1 if NS)

How many children were referred for diagnostic investigation?

____
(-1 if NS)

Total number of confirmed hearing impairments from protocol that fit case definition of this review in this protocol

____
(Specify number or -1=NS)

Total number confirmed to have normal hearing at diagnostic stages of UNHS?

____
(Specify number or -1=NS)

Total number confirmed to have hearing impairment that did not fit review case definition by end of UNHS

____
(Specify number or -1=NS)

Specify reasons for not meeting case definition and numbers in this protocol

____
(Give numbers & reason)

What further information is given on screen negatives after UNHS?

- ☐ None - no further confirmation of normal/abnormal hearing
- ☐ Report of number later confirmed to have normal hearing
- ☐ Report of number later confirmed to have hearing impairment

Report number later confirmed to have normal hearing

____

Report number later confirmed to have hearing impairment fitting the review case definition

____

Report number later confirmed to have hearing impairment not fitting the review case definition

____

Describe reasons provided for changes in hearing status after UNHS

____
(report -1 if not specified)

What further information is given on changes in hearing status for screen positives after UNHS?

- ☐ None - no further confirmation of normal/abnormal hearing
- ☐ Report of number with normal hearing diagnosed later confirmed to have hearing impairment
- ☐ Report of number with hearing impairment later confirmed to have normal hearing

Report number later confirmed to have normal hearing

____

Report number later confirmed to have hearing impairment fitting the review case definition

____

Report number later confirmed to have hearing impairment not fitting the review case definition

____

Describe reasons provided for changes in hearing status after UNHS

____
(report -1 if not specified)

Any notes for this section

____
(-1 if none)

Reported prevalence by study

Does the paper report birth prevalence of hearing impairment at UNHS?

- ☐ No
- ☐ Yes reports prevalence of hearing impairment fitting review case definition
- ☐ Yes reports other prevalences

(Check all that apply)

Report the prevalence & 95% CI of hearing impairment that fit the review case definition (>=26 dB HL, bilateral and non-transient)

____
(Report n per 1000 children or -1 if NS)

What denominator was used?

◯ Whole study population
◯ Sample receiving at least one screening test
◯ Sample reaching the end of screening without drop-out
◯ Unclear
◯ Other
(Report n per 1000 children or -1 if NS)

Any notes, including what 'other' denominator was?

____
(-1 if none)

Report any other prevalences & 95% CIs given in the article

____
(-1 if no other relevant prevalence reported)

Number of children with confirmed hearing impairment fitting case definition of review by age 1 year not detected at UNHS

____
(Report number or -1= NS including UNHS studies without follow-up)

Describe reasons for non-detection if known

____
(Describe - not screened, missed at UNHS, etc.)

Total number of children followed-up after UNHS

____
(Specify number or -1=NS)

Characteristics of children

Is the sex reported of children with HI fitting the review case definition?

◯ Yes
◯ No

Report number of children with HI that were female

____

Number of children with HI that were male

____

Reported sex of all children in study population

◯ Yes
◯ No

Report number of children overall that were female

____

Number of children overall that were male

____

Reported ethnicity of children with HI fitting review case definition?

◯ Yes
◯ No

Report ethnicity categories & number in each category

____

Reported ethnicity for study population?

◯ Yes
◯ No

Report number of children in each ethnicity group reported

____

Characteristics of HI

Are any risk factors for hearing impairment reported separately for children with hearing impairment that fit the case definition of review?

◯ Yes
◯ No
(State no if risk factors were described but could not pick out details for children with bilateral impairment of 26 dB HL or worse)

Report number of hearing impairments with genetic risk factors present (genetic cause identified, family history etc)

____
(Report number or -1=NS)

Report number of hearing impairments with craniofacial abnormalities present

____
(Report number or -1=NS)

Report number of hearing impairments with infectious risk factors present

____
(Report number or -1=NS)

Report number with cytomegalovirus

____
(Report number or -1=NS)

Report number with rubella

____
(Report number or -1=NS)

Report number with other infection

____
(Report number or -1=NS)

Specify other infections present and number

____
(Report number & infection or -1 if NS)

Report number of hearing impairments with perinatal risk factors present: NICU admission, asphyxia or ventilation support requirement, hyperbilirubinaemia, ototoxic medication, other

____
(Report number or -1=NS)

Report number of hearing impairments with NICU admission

____
(Report number or -1=NS)

Report number of hearing impairments with asphyxia and/or ventilation support

____
(Report number or -1=NS)

Report number of hearing impairments with hyperbilirubinaemia

____
(Report number or -1=NS)

Report number of hearing impairments with ototoxic exposure

____
(Report number or -1=NS)

Report number of hearing impairments with other perinatal risk factor

____
(Report number or -1=NS)

Specify perinatal risk factors and numbers

____

Report number of hearing impairments with specified risk factors present that do not fit into above categories

____
(Report number or -1=NS)

Report what other causes were & number in each category?

____

Report number of hearing impairments with no risk factors

____
(Report number or -1=NS)

Report the number of cases fitting the case definition of the review that were SNHL, CHL and mixed separately

____
(-1 if NS)

Report the number of cases fitting the review case definition fitting into the severity groups defined by the paper

____
(-1 if NS)

Quality

Definition of confirmed childhood hearing impairment

- ○ Clearly defined all of the following: minimum severity in dB HL, whether unilateral and bilateral (or just one) cases were included, and whether SNHL/CHL/mixed were included
- ○ Mostly defined (missing maximum 1 criteria on severity in dB HL, inclusion of unilateral/bilateral cases and inclusion of SNHL/CHL/mixed cases)
- ○ Not clearly defined (missing >1 criteria on severity, unilateral/bilateral or anatomy)

Were UNHS methods clearly described?

- ○ Yes (defined at-risk & not at risk protocols, if both relevant, specifying which tests were used & how many times along with pass/fail criteria)
- ○ No or unclear

Did the UNHS protocol clearly describe how high-risk/NICU infants should be managed?

- ○ Yes
- ○ No

Risk of bias in sample - are participants likely to be representative of population?

- ○ No risk of bias identified
- ○ Excluded NICU/at risk infants
- ○ Only included hospital births in a population where these may not be representative of other births
- ○ Took place in hospital(s) with high amount of NICU/neonatal care infants in comparison to normal population
- ○ Other concerns over representativeness

If other risk of bias, specify

____

What was recruitment/population coverage?

- ○ NS/unclear
- ○ At least 95% recruitment rate/population coverage (those receiving at least one screening test divided by total potential study number) as per JCIH, 2007 guidelines
- ○ 90-94% recruitment rate/population coverage
- ○ 80-89% recruitment rate/population coverage
- ○ < 80% recruitment rate/population coverage

What was follow-up of infants through UNHS?

- ◯ NS/unclear
- ◯ At least 90% for (total number referred to further screening after first test minus number lost to follow up) divided by (total number referred for further screening after first test)
- ◯ 80-89% for above equation
- ◯ 70-79% for above equation
- ◯ < 70% for above equation

(>70% fits JCIH guidelines)

Notes

Are there any further relevant references that may have been missed from the search?

____

Any other notes of interest

____
