# Supplementary material for: Prevalence of permanent childhood hearing loss detected at the universal newborn hearing screen: Systematic review and meta-analysis
Source: PLoS One. 2019 Jul 11;14(7):e0219600. doi: 10.1371/journal.pone.0219600 (PMC6622528; doi:10.1371/journal.pone.0219600)
Supplement: S3 File — (DOCX) [file pone.0219600.s006.docx]

***S3 File. QUADAS-2 scoring results***

| **Authors** | **Could the selection of patients have introduced bias?** | **Could the conduct/interpretation of the index test have introduced bias?** | **Could the reference standard, its conduct, or its interpretation have introduced bias?*** | **Could the patient flow have introduced bias?** | **Is there concern that the included patients do not match the review question?** | **Is there concern that the index test, its conduct, or interpretation differ from the review question?** | **Is there concern that the target condition as defined by the reference standard does not match the review question?*** |
| --- | --- | --- | --- | --- | --- | --- | --- |
| Adelola | LOW | HIGH | HIGH | HIGH | LOW | HIGH | HIGH |
| Aidan | HIGH | LOW | HIGH | HIGH | LOW | LOW | HIGH |
| Almenar Latorre | HIGH | LOW | LOW | HIGH | HIGH | LOW | HIGH |
| Antoni | UNCLEAR | HIGH | HIGH | LOW | LOW | LOW | HIGH |
| Bailey | HIGH | UNCLEAR | HIGH | HIGH | HIGH | LOW | HIGH |
| Berninger | UNCLEAR | HIGH | LOW | LOW | UNCLEAR | LOW | HIGH |
| Calcutt | LOW | UNCLEAR | LOW | LOW | LOW | LOW | HIGH |
| Calevo | LOW | LOW | LOW | UNCLEAR | LOW | LOW | HIGH |
| Caluraud | LOW | LOW | HIGH | LOW | LOW | LOW | HIGH |
| Cao-Nguyen | UNCLEAR | UNCLEAR | LOW | LOW | UNCLEAR | UNCLEAR | UNCLEAR |
| De Capua | LOW | LOW | LOW | LOW | LOW | LOW | HIGH |
| Fornoff | LOW | HIGH | HIGH | UNCLEAR | LOW | LOW | HIGH |
| Ghirri | HIGH | LOW | HIGH | UNCLEAR | HIGH | LOW | HIGH |
| Gonzalez | LOW | LOW | HIGH | UNCLEAR | LOW | LOW | HIGH |
| Guastini | UNCLEAR | LOW | HIGH | LOW | UNCLEAR | LOW | HIGH |
| Habib | HIGH | UNCLEAR | HIGH | LOW | HIGH | UNCLEAR | HIGH |
| Magnani | LOW | LOW | HIGH | LOW | LOW | LOW | HIGH |
| Martinez | UNCLEAR | UNCLEAR | HIGH | UNCLEAR | UNCLEAR | LOW | HIGH |
| Mason | HIGH | LOW | HIGH | LOW | HIGH | LOW | HIGH |
| Mehl | UNCLEAR | HIGH | HIGH | UNCLEAR | UNCLEAR | LOW | HIGH |
| Metzger | HIGH | UNCLEAR | HIGH | LOW | HIGH | LOW | HIGH |
| Ng | LOW | LOW | LOW | UNCLEAR | LOW | LOW | HIGH |
| NSW | LOW | UNCLEAR | HIGH | LOW | LOW | LOW | HIGH |
| O'Connor | LOW | UNCLEAR | HIGH | UNCLEAR | LOW | UNCLEAR | HIGH |
| Rohlfs | HIGH | LOW | HIGH | HIGH | HIGH | LOW | HIGH |
| Uilenburg | HIGH | HIGH | HIGH | UNCLEAR | HIGH | UNCLEAR | HIGH |
| Uus | LOW | LOW | HIGH | UNCLEAR | LOW | LOW | HIGH |
| Van der Ploeg | HIGH | UNCLEAR | HIGH | LOW | HIGH | LOW | HIGH |
| Van Kerschaver | LOW | HIGH | HIGH | LOW | LOW | LOW | HIGH |
| Watkin | UNCLEAR | HIGH | LOW | LOW | LOW | LOW | HIGH |
| Wessex | HIGH | HIGH | LOW | LOW | LOW | LOW | HIGH |
| White | UNCLEAR | LOW | HIGH | UNCLEAR | UNCLEAR | LOW | LOW |

*For the question:

Could the reference standard, its conduct, or its interpretation have introduced bias?

LOW= adequate reference standard described with sufficient detail and appropriate methods and follow-up to verify HL status of all children (including screen negatives) after index testing.

HIGH = inadequate reference standard or inadequate methods or follow-up to verify HL status of all children after index testing (including screen negatives)

UNCLEAR = unclear on one or more points

For the question:

Is there concern that the target condition as defined by the reference standard does not match the review question?

LOW=bilateral 26 dB HL permanent HL including sensorineural, conductive, and mixed conditions with details of temporary HL exclusion

HIGH=included unilateral conditions, threshold other than 26 dB HL, included temporary HL, or limited to sensorineural HL - or did not match review question definition for any other reason

UNCLEAR=unclear on one or more aspects of target condition
